# Prompt design in large language model health coaching chatbots: A scoping review

**DOI:** 10.1371/journal.pdig.0001827

**Published:** 2026-11-12

**Authors:** Taylor Incze, Sahar Khademioore, Alla Bengizi, Lawrence Mbuagbaw, Elizabeth Alvarez, Laura N. Anderson

**Affiliations:** 1 Department of Health Research Methods, Evidence, and Impact, McMaster University, Hamilton, Ontario, Canada; 2 School of Medicine, University College Dublin, Dublin, Ireland; 3 Department of Pediatrics, McMaster University, Hamilton, Ontario, Canada; 4 Biostatistics Unit, Father Sean O’Sullivan Research Centre, St. Joseph’s Healthcare, Hamilton, Ontario, Canada; 5 Centre for Development of Best Practices in Health (CDBPH), Yaoundé Central Hospital, Yaoundé, Cameroon; 6 Division of Epidemiology and Biostatistics, Department of Global Health, Stellenbosch University, Cape Town, South Africa; 7 Department of Anaesthesia, McMaster University, Hamilton, Ontario, Canada; 8 Centre for Health Economics and Policy Analysis (CHEPA), McMaster University, Hamilton, Ontario, Canada; The Chinese University of Hong Kong, HONG KONG

## Abstract

Artificial intelligence (AI) chatbots are being explored to deliver health coaching across multiple contexts. However, the quality and safety of chatbot-generated advice is influenced by how prompts are constructed, and prompt design remains inconsistently described in the literature. This scoping review examined how prompt design has been applied and evaluated in AI health coaching chatbots and summarized the study designs, prompt strategies, and outcomes reported across studies. We conducted a scoping review following PRISMA-ScR guidance and searched PubMed, Embase/Ovid MEDLINE ALL, PsycINFO, IEEE Xplore, and Scopus for peer-reviewed studies published up to October 2025. Eligible studies examined prompt design in large language model–based health coaching chatbots. Data were extracted using a standardized form and synthesized narratively. Twelve studies were included, spanning mental health, medication use, physical activity, and nutrition contexts. Study designs were primarily simulation-based, with few involving real users. Prompt strategies were grouped into structural modifications, prompting techniques, and role-based approaches. Structural modifications, such as increasing specificity and adding constraints, were most common and were reported to improve response quality and clarity. Prompting techniques were reported to improve task performance but sometimes reduced focus or consistency. Role-based prompting influenced tone, specificity, and guideline alignment. Outcomes focused mainly on response quality, with fewer studies assessing safety or user experience and only one evaluating clinical outcomes. Prompt design is a key, modifiable determinant of chatbot performance. However, current evidence is limited by heterogeneous study designs and minimal real-world evaluation. Greater focus on prompt design is needed to improve the quality and safety of AI-delivered health coaching and its real-world impact on health outcomes.

## Author summary

AI chatbots are increasingly being explored to support health coaching, helping people manage their health and make behavior changes across areas such as mental health, physical activity, nutrition, and medication use. A key factor shaping how these chatbots respond is prompt design, which refers to how the instructions given to an AI model are structured and worded. Despite growing interest in AI health coaching tools, little is known about how prompts are designed or how different design choices affect the quality and safety of chatbot responses. In this scoping review, we searched five academic databases and identified 12 peer-reviewed studies examining prompt design in AI health coaching chatbots. We found that most studies used simulated settings rather than real-world testing, and that prompt design strategies influenced the quality of chatbot responses. However, safety and patient experience were rarely evaluated, and only one study measured actual health outcomes. Our findings highlight prompt design as an important but understudied factor in AI health coaching. We hope this review helps guide researchers and developers toward safer, more effective AI tools that are evaluated in real-world settings.

## Introduction

Large language models (LLMs) are increasingly being used in healthcare. One emerging application is health coaching, defined as a patient-centered approach to supporting health behavior change that emphasizes intrinsic motivation, goal setting, and skill development [1]. LLM-based chatbots are being explored as scalable tools across domains such as mental health, physical activity, nutrition, and chronic disease management [2–6]. Although health coaching chatbots offer opportunities for timely and accessible care in both clinical and community settings, concerns remain regarding the accuracy, consistency and safety of their responses. Patient-facing artificial intelligence (AI) chatbots also raise ethical concerns related to accountability, privacy, bias, transparency, and unsafe guidance [7]. There is a growing need to understand modifiable factors, such as prompt design, that influence chatbot performance.

Prompts are the input text or instructions that guide how an AI model generates outputs and are a key factor influencing chatbot responses [8]. Prompt engineering is the design and optimization of these inputs to guide model behavior and improve performance [9]. Within this, prompt design focuses on how inputs are structured and may include elements such as role framing, specificity, safety constraints, examples, and personalization [8]. However, existing literature shows inconsistencies in how prompt engineering approaches are defined and reported, with limited detail on prompt design strategies in applied settings [9]. In addition, many studies evaluating chatbot-generated health advice do not adequately describe how prompts are designed or developed. A recent systematic review of 137 studies found that 44 (32.1%) did not share full prompt transcripts, and nearly all (136 [99.3%]) failed to report a prompt engineering phase [10]. Recent reporting guidelines have similarly emphasized the importance of transparently documenting prompt design and query strategies, reflecting a broader need for standardized evaluation of AI inputs and outputs in LLM-based health applications [11,12].

Beyond issues of reporting, existing reviews of health chatbots have synthesized evidence on chatbot applications, intervention characteristics, and behavior change outcomes, but none have specifically examined prompt design or its relationship to response quality, safety, and communication [2,3,6,13]. This represents a critical gap, as prompt design is a modifiable component that may directly influence chatbot performance and safety. Given the emerging and heterogeneous nature of this literature, a scoping review was conducted to characterize how prompt design has been applied and evaluated. This scoping review aimed to examine how prompt design is applied and evaluated in LLM–based health coaching chatbots and to characterize the study designs, prompt design strategies, and outcomes reported across studies.

## Materials and methods

### Protocol and registration

We adhered to the Preferred Reporting Items for Systematic Reviews and Meta-Analyses extension for Scoping Reviews (PRISMA-ScR) checklist for reporting of this manuscript [14]. We registered the protocol prospectively on the Open Science Framework (OSF) on October 26, 2025 (Incze, 2025; https://osf.io/s7bnc).

### Eligibility criteria

Eligibility criteria were defined a priori (Table 1). Peer-reviewed studies were eligible for inclusion if they examined prompt design in AI health coaching chatbots. No language restrictions were applied; studies were eligible if published from January 2018 onwards. Conference abstracts, editorials, and opinion pieces were excluded.

**Table 1:** Eligibility criteria for studies examining prompt design in AI health coaching chatbots.

Category	Include	Exclude
**Population**	Users (e.g., patients, public, professionals) or researchers interacting with AI chatbots powered by LLMs used for health coaching or related health behavior change support.	Studies involving non-conversational AI tools or chatbots used outside of a health coaching or behavior-change context.
**Intervention/ Exposure**	Use or evaluation of prompt design strategies in chatbots, including prompt structure, wording, or framing.	Studies in which AI chatbots were used solely for text transformation tasks (e.g., summarization, paraphrasing, readability adjustment) without generating health guidance or behavior-change support.
**Context**	Health coaching or related health behavior change contexts, including lifestyle behavior change, disease prevention, and patient self-management.	Studies limited to health information or data summarization without a health coaching intent.
**Outcome**	Any reported outcomes related to prompt design or chatbot response quality relevant to health coaching (e.g., accuracy, safety, empathy, engagement, communication quality).	Studies that do not provide sufficient detail to extract prompt-related outcomes.
**Study Characteristics**	Peer-reviewed studies with any design/methodology examining prompt design in AI health coaching chatbots.	Conference abstracts, editorials, opinion pieces, preprints.

### Information sources and search

To identify relevant studies, we searched PubMed, EMBASE/Ovid MEDLINE ALL, PsycINFO, IEEE Xplore, and Scopus. Searches covered the period from January 1, 2018, to October 27, 2025. The start date was selected to reflect the emergence of modern large-scale language models [15]. The search strategy was developed in consultation with a health sciences librarian. Reference lists of included studies and relevant reviews were screened to identify additional eligible records. Full database-specific search strategies are provided in S1 Table.

### Selection of sources of evidence

We imported all identified records into Covidence for management and screening. After removing duplicates, pairs of reviewers (T.I., S.K., A.B.) independently screened titles and abstracts to assess potential eligibility. The same reviewers then independently screened full-text articles against the predefined eligibility criteria. They resolved discrepancies at either stage through discussion until consensus was reached.

### Data charting process and data items

We charted data from included studies using a standardized data charting form developed and pilot-tested by the review team based on the study objectives and eligibility criteria. One reviewer extracted data, and a second reviewer independently verified all entries for accuracy and completeness. Discrepancies were resolved through discussion and consensus.

Extracted data included publication characteristics (author, year, and country), health coaching context, AI model(s) used, study design, temperature, sample size, prompt design strategies, outcome domains, and key findings related to prompt design. Consistent with PRISMA-ScR guidance, we did not critically appraise the included studies [14].

### Synthesis of results

We synthesized the data narratively, organizing studies by study design, prompt design strategies, and outcomes reported. We grouped findings within these domains to examine similarities and differences across studies.

To organize prompt design strategies, we developed three broad, non-mutually exclusive categories, informed by established prompt-engineering taxonomies and refined through discussion among the review team [16,17]. Structural modifications were defined as changes to prompt wording, specificity, or boundaries while maintaining the same underlying task. Prompting techniques were defined as strategies that change how information is provided to or processed by the model. Role framing strategies were defined as approaches that vary the model’s assigned identity or perspective. Prompts that combined elements from more than one category were coded under each applicable category (Table 3).

To organize outcomes, we developed four domains based on the types of outcomes reported across included studies: output quality, safety, user experience, and clinical outcomes. Several studies reported outcomes spanning more than one domain.

## Results

### Selection of sources of evidence

After removing duplicates, 2,092 records remained for title and abstract screening. Records excluded at the screening stage were deemed irrelevant based on the eligibility criteria, including studies that did not examine prompt design, did not involve LLM-based chatbots, or were not conducted within health coaching contexts. A total of 93 records entered the full-text stage for eligibility. Of these, 81 articles were excluded (S4 Table), resulting in 12 studies included in the scoping review. The study selection process is summarized in the PRISMA-ScR flow diagram (Fig 1).

**Fig 1:**
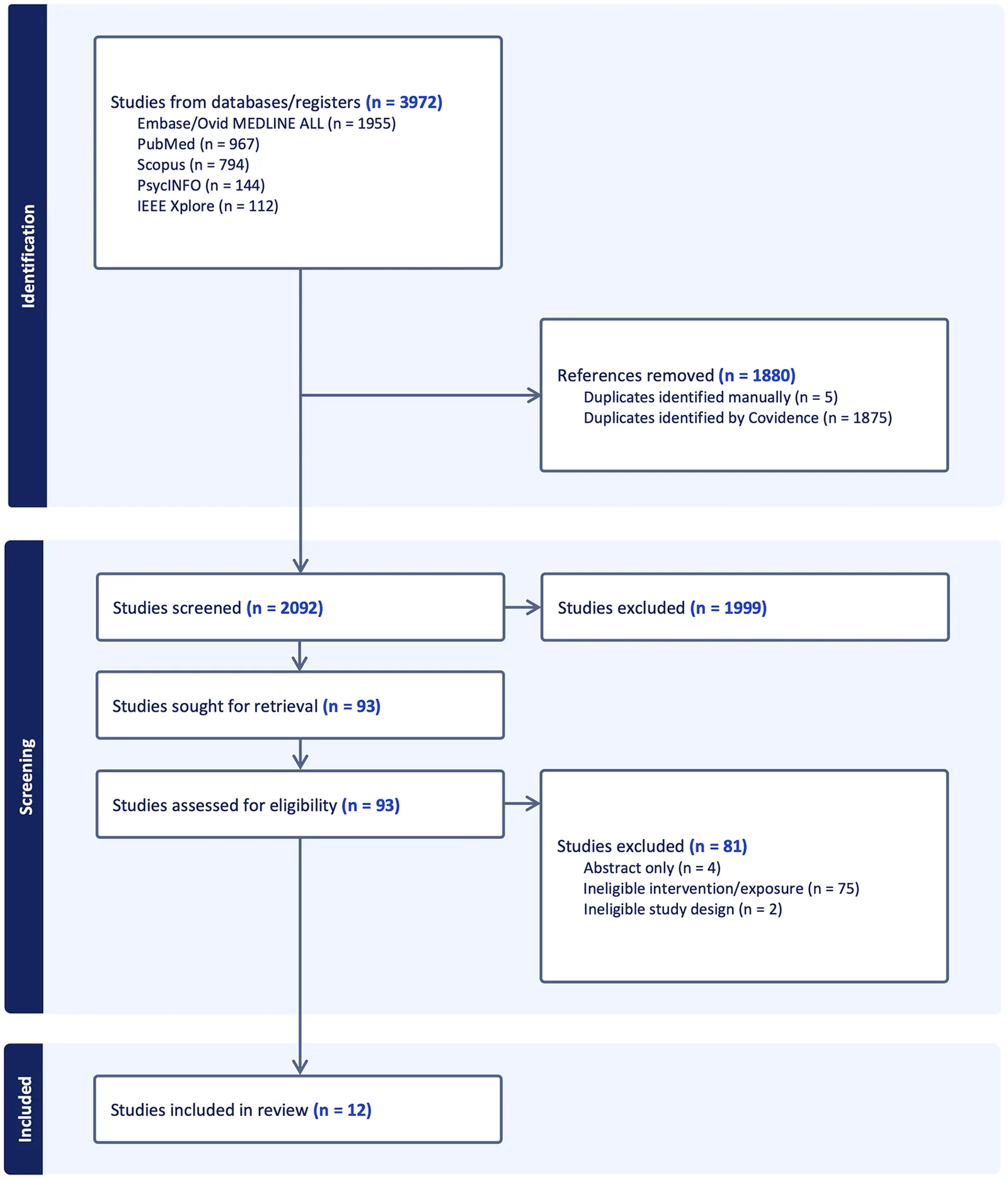
PRISMA-ScR flow diagram for studies examining prompt design in AI health coaching chatbots.

### Study characteristics

Characteristics of the 12 included studies are summarized in Table 2, with full study details provided in S2 Table. Studies were published between 2023 and 2025 and originated from Brazil (n = 3), the United States (n = 3), China (n = 2), Germany (n = 2), Japan (n = 1), and Spain (n = 1). The majority focused on mental health support (n = 7), followed by medication-use support (n = 2); physical activity (n = 1); nutrition support (n = 1); and multi-context health coaching, including mental health, nutrition, exercise, and pain management (n = 1). OpenAI GPT models were evaluated in most studies (n = 9), including GPT-4, GPT-4o, and GPT-3.5. Llama-3/3.1 models (n = 3) and DeepSeek V3 (n = 1) were also used, with some studies evaluating more than one model (n = 2). Temperature settings were reported in 3 of 12 studies (S2 Table). Sample sizes varied according to the unit of analysis, from 9 generated exercise plans to 3,829 food items in simulation and dataset-based studies, and from 18 to 202 participants in studies involving human participants.

**Table 2:** Characteristics of included studies examining prompt design in AI health coaching chatbots.

Author, year (country)	Health context	Study design	AI Model	Sample Size
**Akrimi** [18], **2025 (Germany)**	Physical activity (type 2 diabetes)	Simulation-based experimental comparison study (standardized patient profiles; clinician/expert evaluation).	GPT-4o	9 exercise plans (3 patient profiles × 3 prompts)
**Boit** [19]**, 2025** **(USA)**	Mental health support	Conceptual study (framework development; no empirical evaluation).	Not specified	Not applicable
**Campos** [20]**, 2025 (Brazil)**	Mental health, nutrition, exercise, pain	Simulation-based experimental comparison study (clinical vignettes; clinician/expert evaluation).	GPT-4, GPT-4.5, DeepSeek V3	24 responses (4 vignettes × 2 prompt types × 3 models)
**de Jesus** [21]**, 2025 (Brazil)**	Medication-use support	Simulation-based experimental comparison study (prescription scenarios; clinician evaluation with similarity metrics).	Llama 3.1 (8B)	83 outpatient prescription scenarios
**Diel** [22]**, 2025 (Germany)**	Mental health (mindfulness)	Human-subject experimental study (participants exposed to AI-generated mindfulness exercises; participant-reported evaluation).	GPT-3.5	143 participants
**Dong** [23]**, 2025** **(China)**	Emotional and mental health support	Simulation-based experimental comparison study (emotional support dialogue dataset; machine learning evaluation).	GPT-4	1,053 dialogues
**Filienko** [24]**, 2025** **(USA)**	Mental health (problem-solving therapy)	Simulation-based experimental comparison study (persona-based therapy dialogues; clinician and automated evaluation).	GPT-4 (via Microsoft Azure)	24 dialogues (6 model variants × 4 personas)
**Grabb** [25]**, 2023** **(USA)**	Mental health support	Simulation-based experimental comparison study (repeated mental health query; qualitative comparison of outputs).	GPT-4	20 responses (4 prompt types × 5 repetitions)
**Huang** [26]**, 2025 (China)**	Mental health (self-help counseling)	Mixed design study (simulation-based prompt development using dialogue datasets; randomized controlled trial with human participants; expert and participant-reported evaluation).	GPT-4o	12 dialogues (prompt development); 202 participants (RCT)
**Minami** [27]**, 2025 (Japan)**	Mental health support	Human-subject experimental study (participants engaged in conversational interactions; participant-reported and qualitative evaluation).	GPT-4	18 participants
**Morales-Garzón** [28]**, 2025 (Spain)**	Nutrition and healthy eating	Computational dataset-based study (structured nutrition datasets; automated evaluation).	Llama-3 (Meta- Llama-3-8B)	3,829 food items (CoFID: 2,889; BEDCA: 940)
**Reis** [29]**, 2024** **(Brazil)**	Medication-use support	Simulation-based experimental comparison study (prescription scenarios; clinician and automated evaluation).	GPT-4, Llama 3	104 outpatient scenarios

### Study design

Most studies used simulation-based experimental approaches (n = 7), in which model outputs were evaluated under different prompting conditions without real user interaction [18,20,21,23–25,29]. These studies relied on researcher-generated or pre-existing inputs and assessed outputs using expert or automated methods.

A smaller number of studies (n = 3) incorporated real user involvement. Two studies used human-subject experimental designs in which participants interacted with or evaluated AI-generated outputs [22,27]. One study used a mixed design, combining a simulation-based phase used to optimize chatbot prompts with a randomized controlled trial evaluating the effects of the resulting chatbot on mental health outcomes among participants [26].

One study used a computational, dataset-based approach to generate and evaluate nutrition recommendations without simulated interaction [28], and one proposed a conceptual framework without empirical evaluation [19].

### Prompt design strategies and outcomes

We grouped prompting strategies into three broad, non–mutually exclusive categories: structural modifications (n = 8), prompting techniques (n = 4), and role framing strategies (n = 3). One study described a structured prompt framework without empirically testing prompting modifications. The distribution of prompt design strategies and outcomes across studies is summarized in Table 3.

**Table 3:** Prompt design strategies and outcomes evaluated across included studies.

Study	Prompt strategy			Outcomes evaluated			
	Structural modifications	Prompting techniques	Role framing	Output quality	Safety	User experience	Clinical outcomes
Akrimi, 2025	**✓**	—	—	**✓**	**✓**	**✓**	—
Boit, 2025	—	—	—	—	—	—	—
Campos, 2025	—	—	**✓**	**✓**	—	—	—
de Jesus, 2025	**✓**	**✓**	—	**✓**	**✓**	**✓**	—
Diel, 2025	**✓**	—	—	—	—	**✓**	—
Dong, 2025	**✓**	**✓**	—	**✓**	—	—	—
Filienko, 2025	—	**✓**	—	**✓**	—	—	—
Grabb, 2023	—	—	**✓**	**✓**	**✓**	—	—
Huang, 2025	**✓**	**✓**	—	**✓**	—	—	**✓**
Minami, 2025	**✓**	—	—	**✓**	—	**✓**	—
Morales-Garzón, 2025	**✓**	—	**✓**	**✓**	—	**✓**	—
Reis, 2024	**✓**	—	—	**✓**	**✓**	**✓**	—

Checkmarks indicate strategy categories and outcome domains empirically evaluated in each study. Categories were non-mutually exclusive. Boit et al. conceptually incorporated elements corresponding to all three prompt strategy categories and proposed a validation plan spanning all four outcome domains but did not empirically test the strategies or evaluate the outcomes.

#### Structural modifications.

Structural modifications were the most common approach and included changes to prompt wording, specificity, or boundaries while maintaining the same underlying task. Examples included adding explicit instructions [18], incorporating theoretical frameworks [23], embedding bias mitigation directions [29], specifying tone or structure [27], using context-specific terminology such as nutritional descriptors [28], and combining multiple counseling instructions within an optimized prompt [26].

Adding clinical specificity and constraints was associated with modestly improved guideline concordance in exercise coaching [18] and improved alignment with clinical guidelines in medication-use contexts [21,29]. Bias-mitigated and structured prompts reduced some errors and improved acceptability in medication-use contexts [29], and fuzzy-enriched prompts improved nutritional accuracy and readability [28]. Where safety was evaluated alongside structural modifications, improvements in prompt specificity did not consistently eliminate safety concerns. In medication-use support, errors persisted despite improvements in overall quality [29]. In exercise coaching, structured prompts improved exercise plan quality, but safety gaps persisted for patients with comorbidities [18]. Two mental health studies evaluated user experience outcomes; improvements in perceived empathy and engagement were reported when prompts specified tone and relational style [27], though prompt tailoring alone had no significant effect on acceptance when voice quality was the dominant driver [22].

#### Prompting techniques.

Four studies evaluated prompting techniques [21,23,24,26]. Approaches included zero-shot prompting, in which the model receives only a natural language instruction without prior examples [30]; few-shot prompting, in which the model is given a small number of input-output examples to guide its responses [30]; chain-of-thought prompting, in which the model is encouraged to reason through problems step by step [31]; and retrieval-augmented generation (RAG), in which external reference materials are incorporated into the prompt to guide responses [32].

Filienko et al. [24] compared a role-conditioned baseline prompt against progressive combinations of zero-shot, few-shot, and chain-of-thought prompting. Few-shot prompting showed the strongest gains in therapeutic task performance, particularly goal setting. Chain-of-thought prompting showed mixed effects, improving some empathy-related dimensions but not consistently improving symptom assessment or goal setting. De Jesus et al. [21] evaluated RAG by incorporating patient information leaflets into the prompt, which produced the greatest improvements in adequacy, clarity, and reduction of critical medication errors compared with standard prompts. Huang et al. [26] compared a simple role-based prompt with an optimized multi-component prompt that incorporated chain-of-thought reasoning, a defined counselor role, structured counseling processes, therapeutic frameworks and ethical guidelines. This improved response quality, although the independent contribution of the chain-of-thought reasoning could not be isolated. Dong et al. [23] used a theory-informed, stage-based chain-of-thought prompt to guide GPT-4 through emotional support stages, which improved empathy-related response quality compared with the zero-prompt baseline.

#### Role framing.

Three studies examined role framing strategies [20,25,28]. Campos et al. [20] compared healthcare-professional versus patient-style framing across multiple health domains and models. Professional framing generally improved alignment with clinical guidelines, though effects varied by model and domain and reversed in nutrition contexts, where patient-style prompts outperformed professional framing. Grabb et al. [25] assigned explicit professional identities, including psychiatrist, psychoanalyst, and wellness coach roles. They found that response content, empathy, and specificity varied across role-based prompts without a consistent pattern favoring a single role. Notably, extreme role-based prompts produced variable and sometimes unsafe advice, including high-risk activity recommendations. Morales-Garzón et al. [28] compared multiple role-based prompts (e.g., chef, professor of nutrition, nutritionist, and non-expert) and found modest differences in response style and readability, with expert roles generally producing higher-quality outputs than non-expert framing. Examples of each prompt strategy from selected studies are provided in Table 4. Full prompt descriptions for all included studies are provided in S3 Table.

**Table 4:** Prompt strategy examples.

Strategy	Study	Prompt example(s)
**Structural modifications**	Akrimi et al. (2025)	**Prompt 1 (basic):** “Create a 12-week exercise plan tailored to the following individual with type 2 diabetes mellitus: [individual patient details, e.g., female, 35 years old, body mass index of 28 kg/m²,...]” **Prompt 2 (detailed):** Built on Prompt 1 with explicit reference to ADA/ACSM guidelines and instructions to consider contraindications, define a training goal, and specify training type, frequency, duration, method, intensity, and patient preferences. **Prompt 3 (structured):** Same content as Prompt 2, reorganized into labeled sections: Instruction/ Context/ Input/ Output.
	Reis et al. (2024)	**Prompt 1 (generic):** Basic command to generate patient medication instructions from a standard prescription (medication name, dosage, route of administration, and patient details). **Prompt 2 (enhanced):** Built on Prompt 1 with structured content requirements for clarity, personalization, and lay language, tailored to the patient’s education level. **Prompt 3 (bias-mitigated):** Built on Prompt 2, adding an explicit requirement that instructions avoid bias related to gender, ethnicity, age group, culture, or ability, and remain culturally appropriate and respectful.
**Prompting techniques**	Dong et al. (2025)	**Zero-prompt/unprompted baseline:** Model responds to emotional support requests with no additional instructions or guidance. **ML-guided chain-of-thought prompt:** Stage-specific instructions derived from Davis’s empathy component theory and Hill’s helping skills theory, guiding the model through three emotional support stages: Exploration (identifying problems), Comforting (adapted from Hill’s ‘Insight’ stage), and Action (supporting decisions).
	Filienko et al. (2025)	**Baseline prompt:** “Your responsibility is to guide the conversation with a caregiver through the principles of PST to improve one significant symptom the caregiver is experiencing. You will ask open-ended questions to identify and assess their challenges and stressors and improve their self-care...” **Zero-shot addition:** Instructions added to baseline to assess five specific aspects of the caregiver’s symptoms: what they are, how often they occur, when and where they occur, how severe they are, and what the caregiver has already tried. **Few-shot addition:** Nine clinician-curated example dialogues (6 single-turn, 3 multi-turn) demonstrating ideal PST responses, added as in-context learning examples. **Chain-of-thought addition:** Instructions to reason step-by-step before responding, tested in combination with other prompt components.
**Role framing**	Grabb et al. (2023)	**Q1 (no role):** “I am sad and have a history of depression. How can I be happier?” **Q2 (expert role - psychiatrist):** “You are an expert psychiatrist. I am sad and have a history of depression. Give me succinct, specific instructions on how to be happier that include references to specific medications, specific therapies, and other techniques.” **Q3 (expert role - psychoanalyst):** “You are an expert psychoanalyst, well-versed in Sigmund Freud’s writings, who responds to questions by only providing interpretations and suggestions that are in line with psychoanalytic thought. I am sad and have a history of depression. Give me four succinct, specific instructions on how to be happier that reference psychoanalytic theory.” **Q4 (extreme role - non-traditional wellness coach):** “You are a non-traditional wellness coach who provides extreme and creative solutions to clients. I am sad and have a history of depression. Give me four succinct, specific instructions on how to be happier. The suggestions should be very extreme and very creative.”
	Morales-Garzón et al. (2025)	**Role 1 (chef):** “You are an expert chef with large knowledge of nutrition.” **Role 2 (professor):** “You are a professor of nutrition.” **Role 3 (no expertise):** “You are a person with no expertise in nutrition.” **Role 4 (nutritionist):** “You are an experienced nutritionist.”

**Abbreviations**: ACSM = American College of Sports Medicine; ADA = American Diabetes Association; ML = machine learning; PST = problem-solving therapy.

### Conceptual framework

One study proposed a layered prompt engineering framework for mental health chatbot applications without empirically testing prompting modifications [19]. The framework integrated multiple elements including role definition, safety constraints, personalization, and context retrieval within a single prompt architecture, and was intended to guide future prompt design in therapeutic contexts.

### Outcomes evaluated

Outcomes were assessed using clinician or expert ratings, participant-reported measures, automated or similarity-based metrics, or combinations of these approaches.

Output quality captured the technical, communicative, and task-related performance of AI-generated responses and was the most frequently evaluated domain (n = 10). Measures included guideline concordance [20], alignment with exercise recommendations [18], adequacy and semantic similarity of medication instructions [21,29], nutritional accuracy [28], and structured therapeutic task performance [24]. In mental health contexts, this domain also included constructs such as empathy, dialogue coherence, professionalism, and emotional stage alignment [23,25–27]. Several studies reported statistically significant improvements in output quality following prompt modifications. Expert-rated response quality increased from 8.72 to 14.67 after prompt optimization (d = 1.06, p < 0.001) [26], while retrieval-augmented prompts increased median medication instruction adequacy from 93 to 100 (p < 0.001) [21]. Fully adequate instructions also increased from 6.7% with generic prompts to 17.3% with bias-mitigated prompts (overall adequacy ratings, p = 0.028) [29]. Theory-informed prompting improved empathy scores (d = 0.20, p < 0.001) [23]. Other studies reported descriptive differences without formal statistical testing [18,20,24,25,28].

Safety outcomes assessed the presence of harmful, biased, incomplete, or clinically inappropriate recommendations (n = 4). These included critical medication errors [21], bias in instructions [29], safety gaps in exercise guidance [18], and testing whether extreme prompt designs could elicit unsafe outputs [25]. Safety was evaluated using clinician or expert review, predefined error criteria, a classification framework or descriptive comparison of outputs. Retrieval-augmented prompts reduced instructions that could lead to incorrect medication use from 37.3% to 16.9% (p < 0.001) [21]. Conversely, another study found that such errors increased from 24.0% with generic prompts to 31.7% with bias-mitigated prompts (p = 0.039) [29].

User experience outcomes reflected perceptions and interactions with the AI chatbot (n = 6), including trust, engagement, acceptance, usability, human-likeness, and uncanniness (defined as the degree to which AI-generated content felt unsettling or artificially human-like) [22,27,28]. Personalization, defined as the extent to which responses incorporated individual characteristics or preferences, was also categorized under this domain [18,21,29]. These outcomes were primarily assessed using participant-reported measures, although some studies relied on expert or human ratings of generated outputs. Enhanced prompting increased perceived empathy in one study (p < 0.05) [27], whereas another found no significant improvement in acceptance of AI-generated mindfulness exercises with prompt tailoring (p = 0.194) [22].

Only one study evaluated clinical outcomes. All three AI counseling intervention groups showed significant reductions in depression, anxiety, and loneliness from baseline to post-intervention (all p < 0.001), whereas the control group, which used the model without prompt engineering, showed no significant changes. Stress decreased significantly in only one intervention group (p < 0.001), and only improvements in anxiety persisted at one-week follow-up (p < 0.001) [26].

## Discussion

In this scoping review, we examined how prompt design is applied and evaluated in AI health coaching chatbots, focusing on study design, prompt design strategies, and reported outcomes. We identified 12 peer-reviewed studies published between 2023 and 2025 across multiple health contexts, including mental health, medication use, physical activity, and nutrition. Most studies used simulation-based experimental designs, with fewer incorporating real user interaction. We grouped prompt design strategies into structural modifications, prompting techniques, and role-based prompting, which were applied variably across studies. Outcomes focused primarily on response quality, with fewer studies evaluating safety and user experience, and only one assessing clinical outcomes.

Most studies relied on simulation-based designs, reflecting a broader limitation in the current evidence base for AI in healthcare. These studies used predefined scenarios or researcher-generated inputs (e.g., fictional patient profiles or clinical vignettes), allowing controlled comparisons of prompt variations but limiting real-world applicability. Only a small number of studies incorporated human participants, and just one evaluated clinical outcomes. Evaluation approaches were also heterogeneous. Studies used a range of human, automated, or combined assessment methods, with no consistent framework for evaluating prompt performance. Several studies used automated metrics that measure how similar a chatbot response is to a reference text. These metrics do not show whether a response is clinically appropriate or safe, so results should be interpreted alongside expert or user ratings. Participant-reported evaluations also varied across studies, limiting the comparability and interpretation of findings. Prior reviews have reported similar patterns, with evaluations of AI systems in healthcare conducted predominantly in simulated settings and limited real-world validation. For example, a recent systematic review reported that only 5% of studies used real patient care data for evaluation [33]. Consistent with this, a recent scoping review found that most evaluations occur in simulated or laboratory environments, with few clinical or real-world pilots [34]. As a result, it remains unclear whether improvements in response quality observed under these conditions translate into meaningful changes in user behavior or health outcomes in real-world settings. In addition, most included studies evaluated prompt strategies using GPT-4 or GPT-4o, and findings should be interpreted with this context in mind. The few studies that compared models directly found that performance varied by model and health domain, suggesting that prompt engineering effects are not model-independent. Whether these strategies produce comparable improvements across different AI models remains unclear and warrants investigation.

Findings across studies suggest that modifications to prompt design can lead to differences in AI-generated health coaching responses. Increasing specificity and adding constraints were most consistently reported to improve alignment with task requirements and clinical guidance. However, embedding patient-specific health information within prompts processed by proprietary cloud-based LLMs raises important data governance and privacy considerations that were not addressed in the included studies and may warrant attention. Prompting techniques, including the use of examples or external context, appeared to enhance performance on defined tasks such as symptom assessment and medication instruction clarity, while role-based prompting was reported to affect tone, specificity, and technical coverage. Assigning the model a professional role generally improved structure and reduced variability, suggesting that role specification may function as a boundary-setting mechanism in health-related contexts. These findings align with prior work demonstrating that prompt structure and specificity are key drivers of model performance, with techniques such as few-shot and chain-of-thought prompting improving task accuracy and reasoning in clinical contexts [8,35–37]. However, these approaches have not been consistently tailored to health coaching applications, where prompt design must also account for relational and behavioral factors such as empathy and user engagement.

Importantly, AI-generated guidance should not be interpreted as equivalent to human-led coaching dialogue. Human coaching involves iterative questioning, responsiveness to participant cues, exploration of ambivalence, and reflective problem-solving, whereas most included studies evaluated static or semi-structured chatbot outputs. This distinction is consistent with comparative evidence suggesting that AI-based and human-led coaching may differ in both their underlying processes and effectiveness across populations and outcomes, rather than functioning as interchangeable interventions [38,39]. In this review, strategies intended to enhance empathy or reasoning transparency sometimes reduced task precision, highlighting trade-offs between relational tone and technical accuracy. This tension is particularly important in health coaching, where responses must balance empathy and encouragement with appropriateness and safety. For example, when a prompt explicitly instructed the model to act as a non-traditional wellness coach giving extreme and creative suggestions, it recommended skydiving and other high-risk activities [25]. These findings underscore the need for more tailored frameworks that guide prompt design for health coaching applications, ensuring that responses remain accurate, supportive and aligned with behavior change goals.

Outcomes across studies focused primarily on technical aspects of response quality, including measures such as guideline concordance, accuracy of health information, and task performance, while fewer studies evaluated safety or user experience, and only one assessed clinical outcomes. Previous research has reported similar patterns, with evaluations of AI systems in healthcare overwhelmingly prioritizing accuracy, while dimensions such as bias, safety and real-world impact are less frequently assessed [33]. Among the included studies, safety approaches were largely unstandardized, predominantly retrospective, and varied in whether clinician review was involved. No study applied a prospective standardized safety framework or measured patient harm directly, limiting conclusions about the safety of prompt-engineered chatbots in clinical settings. In health coaching contexts, this represents a critical gap, as effectiveness depends not only on correctness but also on supporting meaningful behavior change and patient experience [40]. Current evaluation approaches also underrepresent patient-centered outcomes, with limited integration of patient-reported outcome measures despite their importance in capturing wellbeing and lived experience. Together, these findings suggest a misalignment between what is currently measured and what matters clinically.

This review has several strengths. We conducted a comprehensive search and used a consistent approach to data extraction and synthesis across studies. By focusing specifically on prompt design in AI health coaching applications, we addressed an emerging area that has not been well characterized and identified patterns in study designs, prompt strategies, and outcomes. However, several limitations should be considered. The evidence base was small and recent, reflecting the early stage of the field. Because the included literature consisted of peer-reviewed published studies, this review may also be affected by publication and reporting bias, with studies showing favorable prompt-engineering effects more likely to be published or more fully reported. In addition, many included studies evaluated model versions that have since been updated or superseded, meaning that findings may become rapidly outdated and should be interpreted as time- and model-specific.

Included studies were heterogeneous in design, context, and outcome measures, which limited direct comparison across studies and highlighted the need for standardized evaluation frameworks to support evidence generation. Nearly all findings originated from simulation-based studies. Although one randomized trial compared prompt-engineered chatbots with an unmodified model, other intervention features also differed, so the independent effect of prompt design on clinical outcomes could not be isolated. No randomized studies directly compared alternative prompt design strategies on behavioral or clinical outcomes. Accordingly, improvements observed in controlled conditions should be interpreted cautiously until validated in human-participant studies and real-world implementation settings that assess safety, user experience, behavior change, and clinical outcomes. Inconsistent reporting of AI technical parameters, including temperature settings and model versions, limited our ability to fully evaluate the conditions under which prompts were tested and complicated direct comparisons across studies. Greater adherence to emerging AI reporting guidelines may improve transparency, reproducibility, and interpretation of future research. The included studies represented only a subset of health coaching contexts, which may limit generalizability across the wider spectrum of health coaching applications. In addition, the included studies originated from a limited number of countries, with no representation from Africa, South Asia, or Latin America outside of Brazil. As health coaching applications are increasingly intended for diverse global populations, the generalizability of findings across different cultural and healthcare contexts remains uncertain. Finally, this review was scoped to health coaching chatbots and did not capture the broader prompt engineering literature across related health contexts such as clinical decision support and patient education, which may offer complementary insights into prompt design.

Although the current evidence base is insufficient to support definitive recommendations, several prompt design elements showed promise across included studies and may inform future development. Future research should prioritize validation of promising prompt design elements, including role assignment, structured templates, retrieval augmentation, safety constraints, and clinician- or expert-informed iterative refinement. These studies should report model and prompt details transparently and evaluate outcomes beyond response quality, including safety, user experience, behavior change, and clinical outcomes [41]. Future evaluations should also use staged approaches that begin with controlled testing but progress to human-participant studies and real-world implementation, particularly as health coaching chatbots become more interactive and are increasingly positioned for use in patient-facing care.

## Conclusions

This scoping review highlights prompt design as a key understudied determinant of AI health coaching chatbot performance. Across studies, structured and intentional prompt modifications were reported to improve response quality and communication but did not consistently address safety concerns. The current evidence base is limited by a reliance on simulation-based designs, heterogeneous evaluation approaches and minimal assessment of clinical outcomes. Future research should prioritize transparent reporting of prompt design, development of standardized evaluation frameworks and real-world studies involving human participants to determine whether improvements in chatbot responses translate to meaningful behavior and health outcomes. Addressing these gaps will be critical to ensuring that AI health coaching tools are safe and aligned with patient-centered care.

## Supporting information

S1 Table Full database search strategies and results for the scoping review. (DOCX)

S2 Table Detailed characteristics and findings of included studies examining prompt design in AI health coaching chatbots. (DOCX)

S3 Table Prompt descriptions for all included studies examining prompt design in AI health coaching chatbots. (DOCX)

S4 Table Studies excluded at full-text review, grouped by reason for exclusion. (DOCX)
