# Supplementary material for: Prompt design in large language model health coaching chatbots: A scoping review
Source: PLOS Digit Health. 2026 Nov 12;5(11):e0001827. doi: 10.1371/journal.pdig.0001827 (PMC13759065; doi:10.1371/journal.pdig.0001827)
Supplement: Supplementary file 1 [file pdig.0001827.s001.docx]

**S1 Table.** Full database search strategies and results for the scoping review

| **Database** | **Search strategy** | **Total results** |
| --- | --- | --- |
| **PubMed** | (("artificial intelligence"[MeSH Terms] OR AI[tiab] OR "large language model*"[tiab] OR chatbot*[tiab] OR "conversational agent*"[tiab] OR "AI assistant*"[tiab] OR "AI model*"[tiab] OR "generative AI"[tiab] OR LLM[tiab] OR GPT[tiab] OR ChatGPT[tiab]) AND ("prompt design"[tiab] OR "prompt engineering"[tiab] OR (prompt[tiab]) OR instruction*[tiab] OR "input formulation"[tiab] OR "query formulation"[tiab] OR "role framing"[tiab] OR "system message*"[tiab] OR "user input*"[tiab] OR "zero-shot"[tiab] OR "few-shot"[tiab] OR "instruction tuning"[tiab]) AND ("health"[tiab] OR "digital health"[tiab] OR "health communication"[tiab] OR "health advice"[tiab] OR "medical advice"[tiab] OR "health coaching"[tiab] OR "health support"[tiab] OR "self-management support"[tiab] OR "health promotion"[tiab] OR "behavior change"[tiab] OR "behaviour change"[tiab] OR "patient education"[tiab]) AND (accuracy[tiab] OR safety[tiab] OR evaluation[tiab] OR framework[tiab] OR principle*[tiab] OR model*[tiab] OR guideline*[tiab] OR recommendation*[tiab] OR reporting[tiab])) AND ("2018/01/01"[Date - Publication] : "3000"[Date - Publication]) | **967** |
| **EMBASE + Ovid MEDLINE ALL**  **(1974 to current)** | 1. exp artificial intelligence/ OR (AI or "large language model*" or chatbot*  or "conversational agent*" or "AI assistant*" or "AI model*"  or "generative AI" or LLM or GPT or ChatGPT).ti,ab.  2. ("prompt design" or "prompt engineering" or prompt or instruction*  or "input formulation" or "query formulation" or "role framing"  or "system message*" or "user input*" or "zero-shot" or "few-shot"  or "instruction tuning").ti,ab.  3. ("health" or "digital health" or "health communication" or "health advice"  or "medical advice" or "health coaching" or "health support"  or "self-management support" or "health promotion" or "behavior change"  or "behaviour change" or "patient education").ti,ab.  4. (accuracy or safety or evaluation or framework or principle*  or model* or guideline* or recommendation* or reporting).ti,ab.  5. 1 and 2 and 3 and 4  6. limit 5 to yr="2018 -Current" | **1,955** |
| **PsycINFO (Ovid)** | 1. (Artificial Intelligence or "large language model*" or chatbot* or "conversational agent*"  or "AI assistant*" or "AI model*" or "generative AI" or LLM or GPT or ChatGPT).ti,ab.  2. exp Artificial Intelligence/ or exp Human-Computer Interaction/ or exp Machine Learning/  3. 1 or 2  4. ("prompt design" or "prompt engineering" or prompt* or instruction* or "input formulation"  or "query formulation" or "role framing" or "system message*" or "user input*"  or "zero-shot" or "few-shot" or "instruction tuning").ti,ab.  5. 3 and 4  6. ("health" or "digital health" or "health communication" or "health advice"  or "medical advice" or "health coaching" or "health support" or "self-management support"  or "health promotion" or "behavior change" or "behaviour change" or "patient education").ti,ab.  7. exp Health Communication/ or exp Health Promotion/ or exp Health Education/ or exp Digital Health/  8. 6 or 7  9. (accuracy or safety or evaluation or framework or principle* or model* or guideline*  or recommendation* or reporting).ti,ab.  10. exp Program Evaluation/ or exp Outcome Assessment/  11. 9 or 10  12. 5 and 8 and 11  13. limit 12 to yr="2018 -Current" | **144** |
| **IEEE Xplore** | ("artificial intelligence" OR "AI" OR "large language model" OR "LLM" OR chatbot OR "conversational agent"  OR "AI assistant" OR "AI model" OR "generative AI" OR GPT OR ChatGPT OR Bard OR Claude OR Gemini)  AND  ("prompt design" OR "prompt engineering" OR "prompt framing" OR "input formulation"  OR "query formulation" OR instruction OR "role framing" OR "system message" OR "user input"  OR "zero-shot" OR "few-shot" OR "instruction tuning" OR "language model prompt" OR "chatbot prompt")  AND  ("health" OR "healthcare" OR "digital health" OR "mHealth" OR "eHealth"  OR "health communication" OR "patient education" OR "behavior change" OR "behaviour change"  OR "health promotion" OR "self-management" OR "health coaching" OR "medical advice" OR "health advice")  AND  (accuracy OR safety OR evaluation OR framework OR guideline OR recommendation OR empathy OR engagement) | **112** |
| **Scopus** | **Document Type:** Journal Articles **Subject Areas:**   - Limited to Medicine - Limited to Computer Science - Limited to Health Professions - Limited to Social Sciences - Limited to Nursing - Limited to Psychology   Search String (all fields)  (TITLE-ABS-KEY("artificial intelligence" OR AI OR "large language model*" OR chatbot* OR "conversational agent*" OR "AI assistant*" OR "AI model*" OR "generative AI" OR LLM OR GPT OR ChatGPT OR Bard OR Claude OR Gemini)) AND (TITLE-ABS-KEY("prompt design" OR "prompt engineering" OR "prompt and framing" OR "input formulation" OR "query formulation" OR instruction* OR "role framing" OR "system message*" OR "user input*" OR "zero-shot" OR "few-shot" OR "instruction tuning") AND (TITLE-ABS-KEY("digital health" OR mhealth OR ehealth OR "health communication" OR "health promotion" OR "preventive health" OR "self-management" OR "behavior change" OR "behaviour change" OR "health coaching" OR "patient education" OR "self care")) AND (TITLE-ABS-KEY(accuracy OR safety OR "communication quality" OR empathy OR engagement OR evaluation OR framework OR guideline OR recommendation OR principle*)) | **794** |

All searches were run on October 27, 2025
