# Supplementary material for: Prompt design in large language model health coaching chatbots: A scoping review
Source: PLOS Digit Health. 2026 Nov 12;5(11):e0001827. doi: 10.1371/journal.pdig.0001827 (PMC13759065; doi:10.1371/journal.pdig.0001827)
Supplement: Supplementary file 2 [file pdig.0001827.s002.docx]

**S2 Table**. Detailed characteristics and findings of included studies examining prompt design in AI health coaching chatbots

| **Author (year, country)** | **Health coaching context** | **AI details: Model/ Temperature/ Sample Size** | **Outcomes evaluated** | **Study design** | **Key findings** |
| --- | --- | --- | --- | --- | --- |
| **Akrimi, 2025 (Germany)** | Physical activity coaching (type 2 diabetes). | GPT-4o /  NR /  9 exercise plans (3 patient profiles × 3 prompts). | Response quality and safety (guideline adherence, progression, feasibility, personalization). | Simulation-based experimental study (standardized patient profiles; clinician/expert evaluation). | Exercise plans showed frequent safety gaps (particularly for patients with comorbidities). Structured prompts improved quality but did not eliminate safety concerns. |
| **Boit, 2025 (USA)** | Mental health support. | Not specified / NA /  NA (conceptual framework). | None (conceptual framework). | Conceptual study (no empirical evaluation). | Proposes a layered prompt engineering framework integrating therapeutic models, personalization, and safety safeguards. |
| **Campos, 2025 (Brazil)** | Mental health, nutrition, exercise, and pain management. | GPT-4, GPT-4.5, DeepSeek V3 /  NR /  24 responses (4 vignettes × 2 prompt types × 3 models). | Guideline concordance (coverage of 89 recommendations). | Simulation-based experimental study (clinical vignettes; clinician/expert evaluation). | Guideline adherence varied by model, prompt type, and domain. Professional prompts improved guideline alignment across most domains and models, while patient prompts performed better in nutrition. |
| **de Jesus, 2025 (Brazil)** | Medication-use support (patient instructions). | Llama 3.1 (8B) / NR / 83 outpatient prescription scenarios. | Response quality (adequacy, clarity, personalization), comparative performance vs reference standard, cosine similarity, and error classification. | Simulation-based experimental study (prescription scenarios; clinician evaluation with similarity metrics). | Prompt refinement improved structure and reduced vague outputs, but retrieval-augmented generation (RAG) produced the greatest improvements in adequacy, clarity, and reduction of critical errors. |
| **Diel, 2025 (Germany)** | Mental health (mindfulness exercises). | GPT-3.5 /  NR /  143 participants. | User acceptance, human-likeness, uncanniness. | Human-subject experimental study (participants exposed to AI-generated content; participant-reported evaluation; n = 143). | Prompt tailoring had no significant effect. Voice quality and contextual fit were the primary drivers of acceptance. |
| **Dong, 2025 (China)** | Emotional and mental health support. | GPT-4 / NR / 1,053 dialogues. | Perceived empathy and emotional support quality. | Simulation-based experimental study (counseling dialogue dataset; machine learning–based evaluation). | Prompting improved empathy. Performance approached human levels for some emotions but remained inferior overall. |
| **Filienko, 2025 (USA)** | Mental health (problem-solving therapy). | GPT-4 / NR / 24 dialogues (6 model variants × 4 personas). | Response quality (symptom assessment, goal setting, empathy, overall therapy quality). | Simulation-based experimental study (persona-based therapy dialogues; clinician and automated evaluation). | Prompting improved therapeutic performance. Few-shot prompting performed best overall and for goal setting; the combined zero-shot/few-shot/CoT model scored highest for symptom assessment. CoT showed mixed effects, with some empathy-related gains but inconsistent task performance. Zero-shot-only models failed preliminary screening and were excluded from full evaluation. |
| **Grabb, 2023 (USA)** | Mental health support. | GPT-4 / NR / 20 responses (4 prompt types × 5 repetitions). | Qualitative response characteristics (specificity, empathy, safety, variability). | Simulation-based experimental study (repeated query; qualitative comparison of outputs). | Prompt design strongly influenced outputs. Role-based prompts improved specificity; extreme prompts produced variable and sometimes unsafe advice. |
| **Huang, 2025 (China)** | Mental health (self-help counseling). | GPT-4o / NR / 12 dialogues (prompt development); 202 participants (RCT). | Response quality and clinical outcomes (depression, anxiety, stress, loneliness). | Mixed design study (simulation-based prompt development using dialogue datasets; randomized controlled trial with human participants; expert and participant-reported evaluation; n = 202). | Prompt optimization improved response quality during development. Participants using the optimized self-help AI counseling system experienced short-term improvements in depression, anxiety, and loneliness. |
| **Minami, 2025 (Japan)** | Mental health support (students). | GPT-4 / 0.7 / 18 participants. | Perceived empathy, understanding, helpfulness, trust. | Human-subject experimental study (participants engaged in conversational interactions; participant-reported and qualitative evaluation; n = 18). | Enhanced prompts improved perceived empathy and engagement but remained inferior to human interactions. |
| **Morales-Garzón, 2025 (Spain)** | Nutrition guidance (food recommendations) | Llama-3 (8B) /  0.0, 0.6, 0.9 / 3,829 food items | Response quality (readability, interpretability), nutritional accuracy, usability. | Computational dataset-based study (nutrition datasets; automated evaluation). | Structured prompts using fuzzy variables improved the quality of recommendations. |
| **Reis, 2024 (Brazil)** | Medication-use support (e-prescribing instructions). | GPT-4, Llama-3 (8B) / 0 (GPT-4); 0.2 (Llama 3) / 104 outpatient scenarios. | Response quality (adequacy, acceptability, personalization), error types, bias. | Simulation-based experimental study (prescription scenarios; clinician and automated evaluation). | Structured and bias-mitigated prompts reduced errors and improved acceptability; errors persisted despite improvements. |

Abbreviations: GPT = Generative Pre-trained Transformer; Llama= Large Language Model Meta AI; RAG = Retrieval-Augmented Generation; CoT = chain-of-thought; NR = not reported; NA = not applicable.
