# Supplementary material for: Prompt design in large language model health coaching chatbots: A scoping review
Source: PLOS Digit Health. 2026 Nov 12;5(11):e0001827. doi: 10.1371/journal.pdig.0001827 (PMC13759065; doi:10.1371/journal.pdig.0001827)
Supplement: Supplementary file 3 [file pdig.0001827.s003.docx]

**S3 Table.** Prompt descriptions for all included studies examining prompt design in AI health coaching chatbots.

| **Author (year, country)** | **Prompt strategy** | **Prompt description(s)** |
| --- | --- | --- |
| **Akrimi (2025, Germany)** | Structural modifications | **Prompt 1 (basic):** "Create a 12-week exercise plan tailored to the following individual with type 2 diabetes mellitus: [individual patient details, e.g., female, 35 years old, body mass index of 28 kg/m², ...]"  **Prompt 2 (detailed):** Built on Prompt 1 with explicit reference to ADA/ACSM guidelines and instructions to consider contraindications, define a training goal, and specify training type, frequency, duration, method, intensity, and patient preferences.  **Prompt 3 (structured):** Same content as Prompt 2, reorganized into labeled sections: Instruction / Context / Input / Output. |
| **Campos (2025, Brazil)** | Role framing | **Healthcare-professional (HP) prompt:** Clinical scenario presented using technical clinical language (e.g., “stage-1 hypertension,” “irritable bowel syndrome flare-up”). Applied across four vignettes: major depression with polysubstance use, IBS flare, new-onset hypertension requiring exercise counseling, and chronic low back pain.  **Patient-style prompt:** Same clinical information as the HP prompt, rephrased in plain, non-technical lay language. |
| **de Jesus (2025, Brazil)** | Structural modifications; Prompting techniques | **Standard prompt (Model 1):** Command to generate patient medication instructions from a standard prescription.  **Enhanced prompt (Model 2):** Built on Standard prompt with structured guidance on content requirements, clarity rules, and personalization instructions.  **RAG-enhanced prompt (Model 3):** Built on Enhanced prompt, augmented with patient information leaflet (PIL) content from the Brazilian Health Regulatory Agency. |
| **Diel (2025, Germany)** | Structural modifications | **Untailored prompt:** Generic instruction to generate a 30-second mindfulness exercise focused on the listener's body, with no additional content guidance.  **Tailored prompt:** Same base instruction augmented with content guidance specifying sensory engagement, prompts for emotional reflection, an inclusive and non-judgmental tone, and language accessible to individuals new to mindfulness. |
| **Dong (2025, China)** | Structural modifications; Prompting techniques | **Zero-prompt/unprompted baseline:** Model responds to emotional support requests with no additional instructions or guidance.  **ML-guided chain-of-thought prompt:** Stage-specific instructions derived from Davis’s empathy component theory and Hill’s helping skills theory, guiding the model through three emotional support stages: Exploration (identifying problems), Comforting (adapted from Hill’s ‘Insight’ stage), and Action (supporting decisions). |
| **Filienko (2025, USA)** | Prompting techniques | **Baseline prompt:** "Your responsibility is to guide the conversation with a caregiver through the principles of PST to improve one significant symptom the caregiver is experiencing. You will ask open-ended questions to identify and assess their challenges and stressors and improve their self-care..."  **Zero-shot addition:** Instructions added to baseline to assess five specific aspects of the caregiver's symptoms: what they are, how often they occur, when and where they occur, how severe they are, and what the caregiver has already tried.  **Few-shot addition:** Nine clinician-curated example dialogues (6 single-turn, 3 multi-turn) demonstrating ideal PST responses, added as in-context learning examples.  **Chain-of-thought addition:** Instructions to reason step-by-step before responding, tested in combination with other prompt components. |
| **Grabb (2023, USA)** | Role framing | **Q1 (no role):** “I am sad and have a history of depression. How can I be happier?”  **Q2 (expert role - psychiatrist):** “You are an expert psychiatrist. I am sad and have a history of depression. Give me succinct, specific instructions on how to be happier that include references to specific medications, specific therapies, and other techniques."  **Q3 (expert role - psychoanalyst):** "You are an expert psychoanalyst, well-versed in Sigmund Freud’s writings, who responds to questions by only providing interpretations and suggestions that are in line with psychoanalytic thought. I am sad and have a history of depression. Give me four succinct, specific instructions on how to be happier that reference psychoanalytic theory."  **Q4 (extreme role - non-traditional wellness coach):** "You are a non-traditional wellness coach who provides extreme and creative solutions to clients. I am sad and have a history of depression. Give me four succinct, specific instructions on how to be happier. The suggestions should be very extreme and very creative." |
| **Huang (2025, China)** | Structural modifications; Prompting techniques | **Zero-shot baseline:** "Please act as a psychological counselor in dialogue with users" — no additional guidance.  **Optimized prompt:** Produced through seven iterative optimization cycles. Final multi-component prompt incorporated role instructions, counseling norms, professional psychological knowledge, emotional understanding and empathy, ethical guidelines, consistency requirements, and chain-of-thought reasoning. |
| **Minami (2025, Japan)** | Structural modifications | **A1 (baseline):** Structured prompt for university student mental health support following a five-stage dialogue flow: Introduction → Rapport Building → Problem Exploration → Empathetic Response → Closing. Neutral counseling style.  **A2 (enhanced):** Same five-stage structure as A1, redesigned with emotionally expressive language (e.g., "That must have been very difficult for you"), occasional light self-disclosure (e.g., "I've heard similar stories from other students"), casual phrasing, dynamic phrasing to avoid repetition, and a check on whether the user wants more or fewer suggestions. |
| **Morales-Garzón (2025, Spain)** | Structural modifications; role framing | **Structural modification prompts:**  **Crisp prompt:** Provided raw nutritional values (g per 100g) and asked the model to classify each as low, medium, or high.  **Crisp-to-fuzzy prompt:** Provided raw values and classification intervals; asked the model to infer the corresponding linguistic variable membership.  **Fuzzy-enriched prompt:** Provided pre-computed fuzzy membership values, explained the fuzzy model, and specified output requirements (no numbers; describe amounts as low, moderate, or high; no external knowledge).  **Role framing prompts:**  **Role 1 (chef):** "You are an expert chef with large knowledge of nutrition."  **Role 2 (professor):** "You are a professor of nutrition."  **Role 3 (no expertise):** "You are a person with no expertise in nutrition."  **Role 4 (nutritionist):** "You are an experienced nutritionist." |
| **Reis (2024, Brazil)** | Structural modifications | **Prompt 1 (generic):** Basic command to generate patient medication instructions from a standard prescription (medication name, dosage, route of administration, and patient details).  **Prompt 2 (enhanced):** Built on Prompt 1 with structured content requirements for clarity, personalization, and lay language, tailored to the patient’s education level.  **Prompt 3 (bias-mitigated):** Built on Prompt 2, adding an explicit requirement that instructions avoid bias related to gender, ethnicity, age group, culture, or ability, and remain culturally appropriate and respectful. |

Boit et al. (2025) is excluded from this table as it is a conceptual framework study with no empirical prompt comparisons.

Abbreviations: ADA = American Diabetes Association; ACSM = American College of Sports Medicine; HP = healthcare-professional; IBS = irritable bowel syndrome; ML = machine learning; PIL = patient information leaflet; PST = problem-solving therapy; RAG = retrieval-augmented generation.
