# Supplementary material for: Prompt design in large language model health coaching chatbots: A scoping review
Source: PLOS Digit Health. 2026 Nov 12;5(11):e0001827. doi: 10.1371/journal.pdig.0001827 (PMC13759065; doi:10.1371/journal.pdig.0001827)
Supplement: Supplementary file 4 [file pdig.0001827.s004.docx]

**S4** **Table.** Studies excluded at full-text review, grouped by reason for exclusion

| **Study** | **Title** |
| --- | --- |
| **Ineligible intervention/exposure (n = 75)** | |
| Abanmy et al., 2025 | Evaluating the accuracy of ChatGPT in delivering patient instructions for medications: an exploratory case study |
| Abate et al., 2025 | Off-the-Shelf Large Language Models for Causality Assessment of Individual Case Safety Reports: A Proof-of-Concept with COVID-19 Vaccines |
| Abou et al., 2024 | ChatGPT vs Expert-Guided Care Pathways for Postesophagectomy Symptom Management |
| Afshar et al., 2024 | Prompt engineering with a large language model to assist providers in responding to patient inquiries: a real-time implementation in the electronic health record |
| Akkan et al., 2025 | Improving readability in AI-generated medical information on fragility fractures: the role of prompt wording on ChatGPT's responses |
| Alapati et al., 2024 | Evaluating insomnia queries from an artificial intelligence chatbot for patient education |
| Alhuzali et al., 2025 | Pre- Trained Language Models for Mental Health: An Empirical Study on Arabic Q&A Classification |
| Ali, 2023 | ChatGPT and Lacrimal Drainage Disorders: Performance and Scope of Improvement |
| Alsabawi et al., 2025 | Readability of custom chatbot vs. GPT-4 responses to otolaryngology-related patient questions |
| CHART Collaborative, 2025 | Reporting guideline for chatbot health advice studies: the Chatbot Assessment Reporting Tool (CHART) statement |
| Armstrong et al., 2024 | Can artificial intelligence (AI) educate your patient? A study to assess overall readability and pharmacists' perception of AI-generated patient education materials |
| Ataguba et al., 2025 | Exploring Large Language Models for Personalized Recipe Generation and Weight-Loss Management |
| Basharat et al., 2025 | ChatGPT and low back pain - Evaluating AI-driven patient education in the context of interventional pain medicine |
| Brown et al., 2024 | Large Language Models: A New Frontier in Pediatric Cataract Patient Education |
| Campbell et al., 2023 | Evaluating ChatGPT responses on obstructive sleep apnea for patient education |
| Campbell et al., 2024 | Evaluating ChatGPT Responses on Thyroid Nodules for Patient Education |
| Chandler et al., 2025 | Exploring ChatGPT's role in women's health self-education: A descriptive study comparing responses with public health guidance |
| Chen et al., 2025 | Enhancing responses from large language models with role-playing prompts: a comparative study on answering frequently asked questions about total knee arthroplasty |
| Chen et al., 2025 | When helpfulness backfires: LLMs and the risk of false medical information due to sycophantic behavior |
| Chiarelli et al., 2024 | Adequacy of prostate cancer prevention and screening recommendations provided by an artificial intelligence-powered large language model |
| Daulat et al., 2025 | Prompt Engineering and Follow-Up Questioning Improves the Readability of Spine Surgery Questions in Large Language Models |
| Delsoz et al., 2025 | Large Language Models: Pioneering New Educational Frontiers in Childhood Myopia |
| Dihan et al., 2024 | Advancing Patient Education in Idiopathic Intracranial Hypertension: The Promise of Large Language Models |
| Dihan et al., 2024 | Large language models: a new frontier in paediatric cataract patient education |
| Dihan et al., 2024 | Using Large Language Models to Generate Educational Materials on Childhood Glaucoma |
| Dihan et al., 2025 | Implementing Generative AI to Enhance Patient Education on Retinopathy of Prematurity |
| Dihan et al., 2025 | Leveraging large language models to improve patient education on dry eye disease |
| Eid et al., 2024 | Optimizing Ophthalmology Patient Education via ChatBot-Generated Materials: Readability Analysis of AI-Generated Patient Education Materials and The American Society of Ophthalmic Plastic and Reconstructive Surgery Patient Brochures |
| Ellison et al., 2025 | De novo generation of colorectal patient educational materials using large language models: Prompt engineering key to improved readability |
| Esmaeilzadeh, 2025 | Ethical implications of using general-purpose LLMs in clinical settings: a comparative analysis of prompt engineering strategies and their impact on patient safety |
| Fenn et al., 2025 | ChatGPT Provides Accurate but Incomplete Responses and Reliably Adjusts Readability to Prompts for Hamstring Injury Frequently Asked Questions |
| Gao et al., 2025 | Large language models: unlocking new potential in patient education for thyroid eye disease |
| Gao et al., 2025 | The use of large language models to enhance cancer clinical trial educational materials |
| Gordon et al., 2024 | Enhancing Patient Communication With Chat-GPT in Radiology: Evaluating the Efficacy and Readability of Answers to Common Imaging-Related Questions |
| Gupta et al., 2025 | Provision of Radiology Reports Simplified with Large Language Models to Patients with Cancer: Impact on Patient Satisfaction |
| Jung et al., 2025 | Prompt engineering with ChatGPT3.5 and GPT4 to improve patient education on retinal diseases |
| Kianian et al., 2024 | The Use of Large Language Models to Generate Education Materials about Uveitis |
| Kim et al., 2024 | Patient-Friendly Discharge Summaries in Korea Based on ChatGPT: Software Development and Validation |
| Kirschenbaum et al., 2025 | Matching Human Expertise: ChatGPT's Performance on Hand Surgery Examinations |
| Kocaballi et al., 2020 | Responses of Conversational Agents to Health and Lifestyle Prompts: Investigation of Appropriateness and Presentation Structures |
| Kufta et al., 2025 | Enhancing Patient Education with AI: A Readability Analysis of AI-Generated Versus American Academy of Ophthalmology Online Patient Education Materials |
| Lambert et al., 2024 | Assessing the Application of Large Language Models in Generating Dermatologic Patient Education Materials According to Reading Level: Qualitative Study |
| Latt et al., 2025 | Evaluation of artificial intelligence (AI) chatbots for providing sexual health information: a consensus study using real-world clinical queries |
| Lautrup et al., 2023 | Heart-to-heart with ChatGPT: the impact of patients consulting AI for cardiovascular health advice |
| Lee et al., 2024 | Evaluating ChatGPT Responses on Atrial Fibrillation for Patient Education |
| Lee et al., 2024 | Impact of Demographic Modifiers on Readabilit of Myopia Education Materials Generated by Large Language Models |
| Lee et al., 2024 | Unlocking Health Literacy: The Ultimate Guide to Hypertension Education From ChatGPT Versus Google Gemini |
| Li et al., 2025 | Benchmarking Large Language Models in Evidence-Based Medicine |
| Li et al., 2025 | Using large language models to generate child-friendly education materials on myopia |
| Liu et al., 2025 | Evaluation of Large Language Models in Tailoring Educational Content for Cancer Survivors and Their Caregivers: Quality Analysis |
| Liu et al., 2025 | Prompt Engineering in Clinical Practice: Tutorial for Clinicians |
| Lu et al., 2025 | Toward fair medical advice: Addressing and mitigating bias in large language model-based healthcare applications |
| Meskó, 2023 | Prompt Engineering as an Important Emerging Skill for Medical Professionals: Tutorial |
| Mishra et al., 2024 | Evaluation of Prompts to Simplify Cardiovascular Disease Information Generated Using a Large Language Model: Cross-Sectional Study |
| Musheyev et al., 2024 | Readability and Information Quality in Cancer Information From a Free vs Paid Chatbot |
| Ni et al., 2024 | Scientific evidence and specific context: leveraging large language models for health fact-checking |
| Olivier et al., 2025 | Assessing ChatGPT responses to patient questions on epidural steroid injections: A comparative study of general vs specific queries |
| Owens et al., 2025 | A comparison of prostate cancer screening information quality on standard and advanced versions of ChatGPT, Google Gemini, and Microsoft Copilot: A cross-sectional study |
| Patel et al., 2025 | Artificial Intelligence in Peripheral Artery Disease Education: A Battle Between ChatGPT and Google Gemini |
| Patel et al., 2025 | Use of ChatGPT for patient education involving HPV-associated oropharyngeal cancer |
| Patil et al., 2024 | Prompt Engineering in Healthcare |
| Qiu et al., 2025 | Enhancing the Readability of Online Pediatric Cataract Education Materials: A Comparative Study of Large Language Models |
| Rebitschek et al., 2025 | Evaluating evidence-based health information from generative AI using a cross-sectional study with laypeople seeking screening information |
| Salmi et al., 2025 | A proof-of-concept study for patient use of open notes with large language models |
| Sina et al., 2024 | Evaluating ChatGPT as a Patient Education Tool for COVID-19-Induced Olfactory Dysfunction |
| Smith, 2025 | Unlocking Large Language Models in PT Education: Harnessing Gen-AI With Strategic Prompt Engineering |
| Sridharan et al., 2024 | Investigating the capabilities of advanced large language models in generating patient instructions and patient educational material |
| Tekin et al., 2025 | ChatGPT-4o as a digital health tool for diabetes technology education: insights on reliability, quality, and readability |
| Vaira et al., 2025 | Enhancing AI Chatbot Responses in Health Care: The SMART Prompt Structure in Head and Neck Surgery |
| Waaler et al., 2025 | Prompt Engineering an Informational Chatbot for Education on Mental Health Using a Multiagent Approach for Enhanced Compliance With Prompt Instructions: Algorithm Development and Validation |
| Warren et al., 2024 | Prompt matters: evaluation of large language model chatbot responses related to Peyronie's disease |
| Warren et al., 2025 | Quality of Chatbot Information Related to Benign Prostatic Hyperplasia |
| Xu et al., 2025 | Assessing the Efficacy of ChatGPT Prompting Strategies in Enhancing Thyroid Cancer Patient Education: A Prospective Study |
| Yan et al., 2024 | Prompt engineering on leveraging large language models in generating response to InBasket messages |
| Yuan et al., 2023 | Advanced prompting as a catalyst: Empowering large language models in the management of gastrointestinal cancers |
| **Conference abstract only (n = 4)** | |
| Abdelnaem et al., 2025 | 045 Implementing generative AI to enhance patient education on retinopathy of prematurity |
| Fereydouni et al., 2024 | Addressing Health-Care Disparities in Patient Comprehension Using GPT-4 |
| Lee et al., 2023 | Evaluating Chatgpt Responses on Atrial Fibrillation for Patient Education |
| Lee et al., 2024 | Racial and Gender Bias in Artificial Intelligence Generated Ophthalmologic Educational Material |
| **Ineligible study design (n = 2)** | |
| Chen et al., 2025 | Leveraging large language models to assist philosophical counseling: prospective techniques, value, and challenges |
| Rebitschek et al., 2025 | Evaluating Evidence-Based Health Risk Communication by Large Language Models: A Cross Sectional Investigation with Laypeople Seeking Cancer Screening Information |
