# Supplementary material for: Prompt design in large language model health coaching chatbots: A scoping review
Source: PLOS Digit Health. 2026 Nov 12;5(11):e0001827. doi: 10.1371/journal.pdig.0001827 (PMC13759065; doi:10.1371/journal.pdig.0001827)
Supplement: Supplementary file 6 [file pdig.0001827.s006.docx]

Response to Reviewer Comments

Prompt design in large language model health coaching chatbots: a scoping review

Manuscript ID: PDIG-D-26-00737R2

Reviewer 3:

Comment:

The reviewer identified several typographical, grammatical, and formatting errors in the revised manuscript, noting that these appeared to result from tracked changes being improperly merged. Specific examples were provided in the Methods, Results, and Discussion sections.

Response:

Thank you for identifying these issues. We reviewed each of the specific examples noted and confirmed that the reported errors appeared only in the tracked-changes version of the manuscript, where some tracked changes had rendered incorrectly. We have regenerated the tracked-changes manuscript so that insertions and deletions are displayed clearly and reviewed the document for any additional formatting or spacing artifacts.
