# Supplementary material for: Prompt design in large language model health coaching chatbots: A scoping review
Source: PLOS Digit Health. 2026 Nov 12;5(11):e0001827. doi: 10.1371/journal.pdig.0001827 (PMC13759065; doi:10.1371/journal.pdig.0001827)
Supplement: Supplementary file 5 [file pdig.0001827.s005.docx]

**Response to Reviewers**

Prompt design in large language model health coaching chatbots: a scoping review

**Reviewer 1**

| **Comments** | **Responses** |
| --- | --- |
| 1. The three prompt strategies (structural modifications, prompting techniques, and role framing) are repetitive and appear in several places. You could consider trimming the redundant descriptions to make the paper flow better. | The formal definitions of all three prompt strategy categories are established once in the Methods section. We have removed the redundant "defined as..." clauses that appeared in the Prompting Techniques and Role Framing subsections of the Results where the terms were unnecessarily re-defined. |
| 2. In Figure 1, 1,999 records were excluded at the title/abstract screening stage without reasons provided. The authors should clarify the main reasons for exclusion to improve transparency and allow readers to assess whether relevant studies were inappropriately omitted. | While Covidence does not capture reason subcategories at the title/abstract screening stage, we have added a sentence to the Selection of Sources of Evidence section (Lines 161-164) clarifying that records excluded at this stage were deemed irrelevant based on the pre-specified eligibility criteria. The manuscript now states: “Records excluded at the screening stage were deemed irrelevant based on the eligibility criteria, including studies that did not examine prompt design, did not involve LLM-based chatbots, or were not conducted within health coaching contexts.” |
| 3. The descriptions of the three prompt strategies are presented entirely in prose. The authors should consider adding a table or figure with concrete prompt examples illustrating each strategy, as this would substantially improve clarity for readers unfamiliar with prompt engineering. | In response, we have added Table 4 (Lines 264-270), which provides illustrative prompt examples organized by strategy category. We have also added Supplementary Table S3, which provides full prompt descriptions for all included studies. |
| 4. The size of the datasets used in these studies are not consistently reported in this scoping review. Since dataset size directly affects the reliability and comparability of the reported findings, the authors should extract and report the number of scenarios, dialogues, or records evaluated in each study where applicable. | We have added a Sample Size column to Table 2, reporting the number of scenarios, dialogues, records, or participants evaluated in each study where applicable. |
| 5. Table 2 currently has limited readability due to frequent line breaks within the 'Prompt design' column. The authors may consider adjusting the column width or font size to improve the visual clarity of this table. | In revising the manuscript, we restructured Table 2 to focus on study characteristics (author/year, health context, study design, AI model, and sample size), removing the Prompt design and Outcomes evaluated columns, which are now captured in a new Table 3 (Lines 205-206) and Table 4 (Lines 264-270). We believe this greatly improves the readability of Table 2. |
| 6. The Discussion touches on some limitations of the included studies but these are scattered and not tied together. It would be stronger if the authors pulled these methodological issues together and connected them directly to the future research directions they propose. Right now those directions feel a bit disconnected from the specific weaknesses found across the studies. | We have revised the Discussion to more explicitly synthesize the methodological limitations of the included studies and connect them to future research directions. Specifically, we note on line 394 that “nearly all findings originated from simulation-based studies, and no randomized studies directly evaluated the effects of different prompt design strategies on behavioral or clinical outcomes.” We further state on line 396 that “improvements observed in controlled conditions should be interpreted cautiously until validated in human-participant studies and real-world implementation settings that assess safety, user experience, behavior change, and clinical outcomes.” We also link heterogeneity in evaluation approaches to the need for standardized evaluation frameworks and inconsistent AI technical reporting to the need for greater transparency and reproducibility in future research. |

**Reviewer 2**

| **Comments** | **Responses** |
| --- | --- |
| 1. Limited Discussion of Risk of Bias and Evidence Quality: Although formal risk-of-bias assessment is not mandatory for scoping reviews, the manuscript does not sufficiently discuss the methodological quality of included studies. The discussion would benefit from a more explicit critique of the strength of evidence underlying the reported findings. For example: Which findings are supported by randomized studies? Which are based solely on simulation? Which findings should be interpreted cautiously? Without such distinctions, readers may overestimate the certainty of conclusions. | We have added text to the Results and Discussion to more explicitly address the strength of evidence underlying our findings. In the Results, we now clarify that nearly all findings originated from simulation-based studies and that no randomized studies directly evaluated the effects of different prompt design strategies on behavioral or clinical outcomes. In the Discussion, we similarly state on line 394 that “nearly all findings originated from simulation-based studies, and no randomized studies directly evaluated the effects of different prompt design strategies on behavioral or clinical outcomes.” We further emphasize on line 396 that “improvements observed in controlled conditions should be interpreted cautiously until validated in human-participant studies and real-world implementation settings that assess safety, user experience, behavior change, and clinical outcomes.” |
| 2. The Search Strategy May Miss Relevant Prompt Engineering Literature. The eligibility criteria focus exclusively on "health coaching chatbots." However, substantial prompt engineering research exists in: Clinical decision support; Medical question answering; Mental health chatbots; Patient education systems; Digital therapeutics. Many of these studies may provide directly relevant evidence regarding prompt design. The authors should justify why the review was restricted to health coaching and discuss how this restriction may have influenced findings. | The decision to focus on health coaching chatbots was intentional, as this context places distinct demands on chatbot responses in terms of behavior change support, empathy, and safety that differ from clinical decision support or medical question answering tasks. We acknowledge, however, that a broader body of prompt engineering literature exists across related health contexts, and have added a sentence to the limitations section (Lines 410-412): “this review was scoped to health coaching chatbots and did not capture the broader prompt engineering literature across related health contexts such as clinical decision support and patient education, which may offer complementary insights into prompt design.” We also note that this scoping review was conducted as part of a broader thesis examining prompt design in health coaching chatbots for behavior change, which further situates and defines the scope of the current work. |
| 3. Insufficient Analysis of Model-Specific Effects: The review notes that GPT models dominated included studies while a few used LLaMA and DeepSeek models. However, the synthesis largely treats prompt engineering effects as model-independent. A discussion of model-specific variability would strengthen the paper. | We have added text to the Discussion (Lines 326-330) acknowledging that the majority of included studies evaluated prompt strategies using GPT-4 or GPT-4o, and that findings should be interpreted with this context in mind. We note that the few studies comparing models directly found that performance varied by model and health domain, suggesting that prompt engineering effects are not model-independent, and that whether these strategies produce comparable improvements across different AI models remains unclear. Specifically, the manuscript states: “In addition, most included studies evaluated prompt strategies using GPT-4 or GPT-4o, and findings should be interpreted with this context in mind. The few studies that compared models directly found that performance varied by model and health domain, suggesting that prompt engineering effects are not model-independent. Whether these strategies produce comparable improvements across different AI models remains unclear and warrants investigation.” |
| 4. Lack of Critical Examination of Safety Evaluations: The manuscript repeatedly notes that safety was assessed in only four studies. However, safety evaluation itself is not critically examined. Important questions remain unanswered: Were safety frameworks standardized? Were harms assessed prospectively or retrospectively? Were evaluations performed by clinicians? Were high-risk scenarios tested? | We have expanded the Discussion to more critically examine how safety was evaluated across the included studies. Specifically, we now state on line 368 that “safety approaches were largely unstandardized, predominantly retrospective and varied in whether clinician review was involved.” We also note on line 370 that “no study applied a prospective standardized safety framework or measured patient harm directly, limiting conclusions about the safety of prompt-engineered chatbots in clinical settings.” |
| 5. A more detailed discussion is warranted because safety remains one of the major barriers to clinical deployment of LLMs. The review concludes that prompt design matters, but it stops short of providing actionable recommendations. Readers would benefit from a synthesis of emerging best practices such as: Explicit role assignment; Safety constraints; Structured prompt templates; Retrieval augmentation; Prompt transparency reporting. A practical summary table would increase the translational value of the review. | We have added text to the Discussion synthesizing prompt design elements that showed promise across included studies. Specifically, we state on line 415 that “future research should prioritize validation of promising prompt design elements, including role assignment, structured templates, retrieval augmentation, safety constraints, and clinician- or expert-informed iterative refinement.” We have framed these as elements warranting further investigation rather than established best practices, given the limited and heterogeneous evidence base. We present these elements narratively rather than as formal recommendations in a summary table to avoid implying a level of certainty or standardization not yet supported by the evidence. |
| 6. The manuscript reports country distributions but does not discuss implications. The absence of studies from Africa, South Asia, and Latin America (outside Brazil) should be highlighted because health coaching applications are often intended for diverse populations. | We have added a sentence to the limitations section (Lines 406-409) noting that included studies originated from a limited number of countries and acknowledging that the generalizability of findings across different cultural and healthcare contexts remains uncertain. The manuscript now states: “the included studies originated from a limited number of countries, with no representation from Africa, South Asia, or Latin America outside of Brazil. As health coaching applications are increasingly intended for diverse global populations, the generalizability of findings across different cultural and healthcare contexts remains uncertain.” |

**Reviewer 3**

| **Comments** | **Responses** |
| --- | --- |
| 1. Major Weaknesses: The primary deficiency of this scoping review is the absence of a standardized assessment of artificial intelligence (AI) reporting quality among the included studies. For a review explicitly focused on "prompt design," it is critical to evaluate whether the primary literature adequately reported large language model (LLM) hyperparameters, exact model versions, and full prompt architectures. The extraction methodology lacks a comprehensive, multi-item reporting checklist to verify the scientific rigor and reproducibility of the underlying data. Furthermore, the categorization of prompt strategies was developed solely through internal discussion without relying on established computational linguistic or machine learning taxonomies. | Although formal quality appraisal is not typically required for scoping reviews, as scoping reviews aim to map the breadth of available evidence rather than evaluate the strength of effect or association (Munn et al., 2018), we agree that transparent reporting of AI parameters and prompt details is important for reproducibility. We therefore expanded our data charting to better capture model version, temperature settings, and sample size in Supplementary Table S2, with full prompt details for each included study added to Supplementary Table S3. We also revised the Methods section to clarify that our prompt strategy taxonomy was informed by established prompt-engineering literature. In the Discussion, on line 399 we now state that “inconsistent reporting of AI technical parameters, including temperature settings and model versions, limited our ability to fully evaluate the conditions under which prompts were tested and complicated direct comparisons across studies.” |
| 2. Minor Weaknesses: There are several typographical errors in the author affiliations section. The PRISMA flow diagram and corresponding text lack detailed stratification of exclusion criteria. Additionally, there is a minor chronological discrepancy regarding the protocol registration and search execution dates that requires clarification. | We have reviewed the author affiliation formatting, clarified the prospective registration and search timeline, and expanded the Selection of Sources of Evidence section to improve transparency regarding exclusion reasons (See detailed comments below). |
| Specific Comment 1. Page 1, Lines 5-6: The author affiliations contain typographical errors, specifically the use of periods and missing commas (e.g., "1.8" and "18" instead of superscript "1, 8"). | We have reviewed the author affiliation formatting (Lines 5-6) to ensure affiliations are properly separated and consistently formatted throughout. |
| 3. Page 3, Line 79: The discussion of risks and safety concerns of chatbots is overly narrow. The context must be broadened to include the systemic risks and ethical dilemmas of AI chatbot integration in scientific and healthcare applications. Integrate the following reference to support this expansion: [Ben Saad H, Dergaa I, et al. The assisted Technology dilemma: a reflection on AI chatbots use and risks while reshaping the peer review process in scientific research. AI & SOCIETY 2025, 40(7):5649-5656.] | We have broadened the safety discussion to acknowledge ethical concerns related to accountability, privacy, bias, transparency, and unsafe guidance in patient-facing health coaching chatbots (Lines 79-80), citing Fareed et al. (2025) (“A systematic review of ethical considerations of large language models in healthcare and medicine,” Front Digit Health, 2025;7, doi:10.3389/fdgth.2025.1653631). Ben Saad et al. (2025) focuses on AI use in academic peer review, which differs from the patient-facing health coaching context of our review; we therefore cited Fareed et al. in its place. Specifically, we added: “Patient-facing AI chatbots also raise ethical concerns related to accountability, privacy, bias, transparency, and unsafe guidance.” |
| 4. Page 4, Lines 90-93: The assertion regarding the lack of transparent reporting of prompt design misses a broader, critical point: the general lack of standardized evaluation for AI inputs and outputs across health and performance technologies. Note the calls for standardized assessments of AI tools to support the argument for structured prompt evaluation, referencing: [Dhahbi W, Chamari K: The Algorithmic Athlete: A Call to Standardize Assessment of Sensor Technologies and Artificial Intelligence. International Journal of Sports Physiology and Performance 2026.] | We agree that transparent prompt reporting should be situated within the broader need for standardized evaluation of AI inputs and outputs. We have combined this point with the adjacent sentence on reporting guidelines so it is directly supported by citation, rather than standing alone. The manuscript now reads: “Recent reporting guidelines have similarly emphasized the importance of transparently documenting prompt design and query strategies, reflecting a broader need for standardized evaluation of AI inputs and outputs in LLM-based health applications,” citing Huo et al. (2025) (“Reporting Guideline for Chatbot Health Advice Studies,” JAMA Netw Open) and Gallifant et al. (2025) (“The TRIPOD-LLM reporting guideline for studies using large language models,” Nat Med) (Lines 87-97). Since, Dhahbi & Chamari (2026) focuses on wearable sensor technologies and AI assessment frameworks in sport science, which we feel differs from the LLM-based health coaching chatbot context of our review, we have not cited this reference. |
| 5. Page 5, Lines 108-109 & Line 121: There is a chronological inconsistency regarding the prospective registration date of "October 26, 2025" and the search execution date of "October 27, 2025". Clarify how the protocol was adequately prospective if the database search was conducted the subsequent day. | The protocol was registered on October 26, 2025, and the database search was conducted on October 27, 2025. The protocol was finalized and publicly registered before any data were retrieved, and no changes were made to the protocol after the search was initiated. We have clarified this sequence in the Methods section (Line 113). The manuscript states: “We registered the protocol prospectively on the Open Science Framework (OSF) on October 26, 2025…” and separately, “Searches covered the period from January 1, 2018, to October 27, 2025.” |
| 6. Page 7, Lines 137-139: The data charting process fails to extract critical technical parameters essential for AI reproducibility. The methodology must incorporate a comprehensive reporting checklist to capture whether the included studies detailed exact LLM versions, temperature settings, context window limits, and prompt token counts. | We have expanded data extraction to capture AI technical reporting characteristics that were feasible to extract across included studies, including model version, temperature settings, and sample size, now reported in Supplementary Table S2. We focused on the technical parameters most consistently available and added inconsistent AI technical reporting as a limitation in the Discussion, noting that “inconsistent reporting of AI technical parameters, including temperature settings and model versions, limited our ability to fully evaluate the conditions under which prompts were tested and complicated direct comparisons across studies.” (Lines 399-402) |
| 7. Page 8, Lines 144-149: The conceptual categories for prompt design strategies (structural modifications, prompting techniques, role framing) are not grounded in established machine learning literature. Provide a theoretical justification or cite existing computer science frameworks to validate this taxonomy. | We have revised the Methods section (Lines 148-150) to provide a clearer rationale for our taxonomy of prompt design strategies. Specifically, we now clarify how the categories were informed by established prompt-engineering literature and refined through discussion among the review team. We also added relevant prompt-engineering survey and taxonomy references to support categories related to prompt structure, prompting techniques, and role framing. Specifically, the manuscript now states: “To organize prompt design strategies, we developed three broad, non-mutually exclusive categories, informed by established prompt-engineering taxonomies and refined through discussion among the review team.” (references 16–17: Schulhoff et al., 2025, The Prompt Report: A Systematic Survey of Prompt Engineering Techniques; Liu et al., 2023, Pre-train, Prompt, and Predict: A Systematic Survey of Prompting Methods in Natural Language Processing, ACM Comput Surv.) |
| 8. Page 10, Lines 159-160: The PRISMA flow diagram groups 75 excluded studies under a single "Wrong intervention" label. Stratify this number to specify how many were excluded for lacking LLMs, lacking a chatbot interface, or lacking the health coaching element to improve methodological transparency. | We appreciate the reviewer’s suggestion. “Wrong intervention” was a predefined full-text exclusion category in Covidence, and the reasons within this category were not further subcategorized during screening. We therefore could not reliably retrospectively stratify the 75 records into mutually exclusive counts for studies lacking an LLM, a chatbot interface, or a health coaching element. To improve clarity and avoid implying that the interventions themselves were incorrect, we have relabeled this category as “Ineligible intervention/exposure” in the revised PRISMA flow diagram (Fig 1). |
| 9. Page 11-14, Table 2: The "Prompt design" column provides generic summaries rather than actionable data. Extract and present specific verbatim prompt examples or structural templates from the primary studies to improve the utility of this synthesis. | In revising the manuscript, we restructured Table 2 (Lines 182-183) to focus on study characteristics, removing the Prompt design column. We have now added Table 4 (Lines 264-270), which provides verbatim prompt examples organized by strategy category, and Supplementary Table S3, which contains full prompt descriptions for all included studies. |
| 10. Page 17, Lines 212-217: The definitions of specific prompting techniques (e.g., zero-shot, few-shot, chain-of-thought, retrieval-augmented generation) lack supporting citations from the foundational AI literature. Integrate primary computational references for these concepts. | We have added citations to the foundational literature for each technique where first introduced in the Results section (Lines 229-235): zero-shot and few-shot prompting cite reference 30 (Brown et al., 2020, “Language Models are Few-Shot Learners”), chain-of-thought prompting cites reference 31 (Wei et al., 2023, “Chain-of-Thought Prompting Elicits Reasoning in Large Language Models”), and retrieval-augmented generation cites reference 32 (Lewis et al., 2021, “Retrieval-Augmented Generation for Knowledge-Intensive NLP Tasks”). The manuscript states: “Approaches included zero-shot prompting, in which the model receives only a natural language instruction without prior examples; few-shot prompting, in which the model is given a small number of input-output examples to guide its responses; chain-of-thought prompting, in which the model is encouraged to reason through problems step by step; and retrieval-augmented generation (RAG), in which external reference materials are incorporated into the prompt to guide responses.” |
| 11. Page 21, Lines 294-298: The discussion of structural modifications omits an analysis of data privacy and data governance. Address the implications of embedding sensitive physiological/behavioral parameters or strict clinical guidelines within prompts processed by proprietary, cloud-based LLMs. | We have added a sentence to the Discussion (Lines 334-336) acknowledging that embedding patient-specific information within prompts processed by proprietary cloud-based LLMs raises data governance and privacy considerations that were not addressed in the included studies. The manuscript now states: “embedding patient-specific health information within prompts processed by proprietary cloud-based LLMs raises important data governance and privacy considerations that were not addressed in the included studies and may warrant attention.” |
| 12. Page 21, Lines 297-302: The comparison between structural modifications and prompting techniques for health coaching lacks depth regarding how AI-generated guidance contrasts with actual human-led dialogue. Contrast these findings with existing literature evaluating AI-based guidance against human coaching. Cite the following reference: [Sahli H et al. Comparing human-led reflective dialogue and AI-based tactical guidance as innovative training approaches in soccer. BMC Sports Science, Medicine and Rehabilitation 2026, 18(1):246.] | We have expanded the Discussion (Lines 348-361) to clarify that improvements in AI-generated responses observed in prompt-engineering studies should not be interpreted as equivalent to human-led coaching dialogue. We also added literature directly comparing AI-based and human-led coaching. Terblanche et al. (2022) compared goal-attainment outcomes across two longitudinal randomized studies involving AI chatbot and human coaching and found similar goal-attainment outcomes, although the authors suggested that the two approaches operated through different processes. De Haan et al. (2026) conducted a direct randomized controlled comparison and found that human coaching produced greater improvements than AI chatbot coaching across most evaluated outcomes. Together, these studies support the conclusion that AI-based guidance and human-led coaching may differ in their processes and effectiveness across contexts, populations, and outcomes rather than functioning as interchangeable interventions. We considered the suggested study by Sahli et al. (2026); however, it examines tactical guidance within soccer training rather than health coaching or patient-facing behavioral support. We therefore selected comparative studies more directly aligned with the coaching context and research question of our review. Specifically, the manuscript now states: “AI-generated guidance should not be interpreted as equivalent to human-led coaching dialogue… This distinction is consistent with comparative evidence suggesting that AI-based and human-led coaching may differ in both their underlying processes and effectiveness across populations and outcomes, rather than functioning as interchangeable interventions.” |
| 13. Page 23, Lines 338-339: The limitations section does not address the high risk of publication bias and the rapid obsolescence of findings. Address this limitation directly, as the majority of the included literature evaluates models that are rapidly superseded by newer architectures. | We have revised the limitations section (Lines 384-390) to directly address both potential publication/reporting bias and the rapid obsolescence of findings in this fast-moving area. Specifically, we now note that the review may be affected by publication and reporting bias because studies showing favorable prompt-engineering effects may be more likely to be published or fully reported. We also clarify that many included studies evaluated model versions that have since been updated or superseded, meaning that findings should be interpreted as time- and model-specific. The manuscript now states: “Because the included literature consisted of peer-reviewed published studies, this review may also be affected by publication and reporting bias, with studies showing favorable prompt-engineering effects more likely to be published or more fully reported. In addition, many included studies evaluated model versions that have since been updated or superseded, meaning that findings may become rapidly outdated and should be interpreted as time- and model-specific.” |
| 14. Page 23, Lines 346-349: The conclusion's call for standardized evaluation frameworks and real-world studies is vague. Provide actionable direction by recommending the adoption of modular frameworks for the development and validation of autonomous AI agents to ensure safety and clinical alignment. Support this recommendation by citing: [Dergaa I et al. Toward autonomous artificial intelligence agents in sports science: a modular framework. Biology of Sport 2026, 43(1).] | We have revised the Discussion and Conclusion (Lines 415-422) to provide more actionable direction for future research. Specifically, we now recommend transparent reporting of prompt and model details, the use of structured and standardized evaluation frameworks, staged validation progressing from controlled testing to human-participant and real-world studies, and assessment of outcomes beyond response quality, including safety, user experience, behavior change, and clinical outcomes. We considered the suggested reference by Dergaa et al. (2026); however, it addresses autonomous AI agents in sports science rather than patient-facing health coaching chatbots. We therefore selected a framework more directly aligned with the scope of our review. We now cite Hua et al. (2025) (“Standardizing and Scaffolding Health Care AI-Chatbot Evaluation: Systematic Review,” JMIR AI), a hierarchical, stakeholder-informed framework that prioritizes safety, privacy and fairness; trustworthiness and usefulness; and design and operational effectiveness. This citation has been added to the Discussion to support our recommendation for standardized and clinically relevant evaluation of health care chatbots. The manuscript states: “Future research should prioritize validation of promising prompt design elements, including role assignment, structured templates, retrieval augmentation, safety constraints, and clinician- or expert-informed iterative refinement… Future evaluations should also use staged approaches that begin with controlled testing but progress to human-participant studies and real-world implementation.” |
