# Supplementary material for: Prompt design in large language model health coaching chatbots: A scoping review
Source: PLOS Digit Health. 2026 Nov 12;5(11):e0001827. doi: 10.1371/journal.pdig.0001827 (PMC13759065; doi:10.1371/journal.pdig.0001827)
Supplement: Supplementary file 7 [file pdig.0001827.s007.docx]

**Response to Reviewers**

Prompt design in large language model health coaching chatbots: a scoping review

Manuscript ID: PDIG-D-26-00737R3

**Reviewer 3**

| **Comments** | **Responses** |
| --- | --- |
| 1. **General Comments**  The manuscript presents a scoping review examining prompt design strategies in large language model health coaching chatbots. A major weakness of the manuscript is the absence of a structured quality appraisal of the included studies. While optional in scoping reviews, critical appraisal is strictly necessary when assessing experimental artificial intelligence applications in clinical or sports medicine contexts to distinguish between rigorous methodological frameworks and anecdotal observations. | Thank you for this comment. Our scoping review aimed to describe how prompt design has been applied and evaluated in LLM-based health coaching chatbots and to characterize the study designs, prompting strategies, and outcomes reported, rather than to appraise individual studies or estimate pooled effects. Critical appraisal is optional in scoping reviews (Munn et al., 2018; Peters et al., 2020). We have clarified this in the Methods (lines 141–142): “Consistent with PRISMA-ScR guidance, we did not critically appraise the included studies.” We also discuss the predominance of simulation-based evaluations and limited real-world validation as important limitations when interpreting the findings (lines 404–411). |
| 2. The synthesis relies heavily on the descriptive categorization of structural modifications, prompting techniques, and role framing, yet it fails to rigorously map how these prompt architectures interact with specific physiological or behavioral outcomes. | The outcomes evaluated in each included study are summarized in Table 3. Only one study evaluated clinical outcomes (lines 307–311). Its randomized trial compared prompt-engineered AI counseling interventions with an unmodified model, but other intervention features also differed, so the independent effect of prompt design on clinical outcomes could not be isolated. None of the included studies measured physiological or behavioral outcomes, or directly compared alternative prompt design strategies on behavioral or clinical outcomes. We highlight these limitations and the need for further research in the Discussion (lines 404–411 and 425–434). |
| 3. A minor weakness is the inconsistent integration of extracted variables. The methodology states that technical parameters such as model temperature were extracted, but these variables are largely absent from the results synthesis and study characteristics table. | We have added summaries of temperature reporting and sample sizes to the Results (lines 179–182), rather than presenting these variables only in the tables. Temperature settings were reported in 3 of 12 studies, and sample sizes are described according to the unit of analysis. Per-study information remains available in Table 2 and S2 Table. Please also see our responses to comments 7 and 10. |
| 4. Scientific Content, Methodology, and Statistics  The methodological framework adheres to the PRISMA-ScR guidelines and includes a prospectively registered protocol. The search strategy encompasses appropriate databases for the intersection of computer science and health, including IEEE Xplore and PubMed. The authors correctly omitted statistical meta-analysis due to the heterogeneity of the 12 included simulation-based studies. The narrative synthesis lacks a professional biomedical engineering or biostatistical perspective on how natural language processing evaluation metrics are quantified. The categorization of outcomes into output quality, safety, user experience, and clinical outcomes is logical, but the manuscript does not critically evaluate the statistical validity of the automated similarity metrics or participant-reported measures utilized in the primary studies. Relying on superficial descriptions of evaluation metrics limits the academic rigor required for artificial intelligence integration in health behavior change protocols. | Thank you for highlighting several strengths of our study. We have expanded the “Outcomes Evaluated” section of the Results (lines 282–311) to report the direction, magnitude, and statistical significance of relevant comparisons where available. The additions describe improvements in output quality, mixed findings for medication-related safety, both significant and nonsignificant user experience results, and clinical outcomes from the one randomized trial. We also distinguish studies that reported descriptive comparisons without formal statistical testing. To clarify the limitations of the evaluation approaches, we added the following to the Discussion (lines 328–331): “Several studies used automated metrics that measure how similar a chatbot response is to a reference text. These metrics do not show whether a response is clinically appropriate or safe, so results should be interpreted alongside expert or user ratings.” Our aim is to describe and contextualize the evaluation methods used in the included studies, rather than independently validate those measures or estimate pooled effects. |
| 5. Page 5, Line 106: The introduction abruptly concludes the rationale for the scoping review without contextualizing how artificial intelligence prompt design methodologies compare to broader automated applications in health and personalized prevention. Broaden the discussion on methodological frameworks used in scoping reviews within health contexts to provide a comparative baseline for evaluating structural modifications. Include the following reference to strengthen this context: [Dhahbi W, Jebabli N, Souaifi M, Ceylan Hİ, Ben Saad H, Chamari K, Pyne DB, Chaabene H: Application of Artificial Intelligence for Predicting Sports Injuries and Customizing Personalized Prevention Strategies: A Scoping Review. Bioengineering 2026, 13(6):692.]. | The Introduction already situates prompt design within the broader need for standardized evaluation of AI health applications (lines 93–95) and summarizes prior reviews of health chatbots (lines 97–100). The suggested review by Dhahbi et al. (2026) addresses machine learning for sports injury prediction and personalized prevention, rather than prompt design in LLM-based health coaching chatbots. We have therefore not added this reference. |
| 6. Page 7, Lines 123-124: The search period is defined as extending to October 27, 2025. The reference list includes publications dated 2026, such as Njei et al., 2026, and de Haan et al., 2026. Correct the chronological boundaries of the search strategy and align the inclusion dates with the bibliography. | The October 27, 2025 cutoff applies to the search for eligible studies, not to all publications cited for context in the manuscript. All 12 included studies were published between 2023 and 2025 (Table 2). Njei et al. (2026) and de Haan et al. (2026) are cited in the Discussion as contextual literature and were not included in the evidence synthesis. |
| 7. Page 7, Lines 137-142: The data charting process explicitly extracts “temperature” and “sample size”. These critical technical metrics are excluded from the primary results text and Table 2. Integrate all charted artificial intelligence parameters into the final synthesis to ensure reproducibility. | Sample size is reported for each study in the final column of Table 2, while temperature settings and other technical details are presented in S2 Table. We have added the following summary to the Results (lines 179–182): “Temperature settings were reported in 3 of 12 studies (S2 Table). Sample sizes varied according to the unit of analysis, from 9 generated exercise plans to 3,829 food items in simulation and dataset-based studies, and from 18 to 202 participants in studies involving human participants.” |
| 8. Page 8, Lines 148-154: The taxonomy of prompt design strategies defines structural modifications, prompting techniques, and role framing. The methodology lacks a transparent mapping of how complex prompts containing multiple overlapping elements were isolated and categorized. Provide a clear methodological protocol for handling hybrid prompt designs. | The three prompt-design categories were intended to be non-mutually exclusive. We have clarified how hybrid prompts were classified in the Methods (lines 153–154): “Prompts that combined elements from more than one category were coded under each applicable category (Table 3).” This classification captures overlapping components without assuming that their independent effects can be isolated from the included studies. |
| 9. Page 9, Line 165: The PRISMA flow diagram indicates 81 full-text articles were excluded. The manuscript fails to provide a categorized breakdown of exclusion reasons beyond the broad numerical summary in the diagram. Add a supplementary table detailing the specific rationales for full-text exclusions. | We have added S4 Table, which lists all 81 full-text articles excluded after screening, grouped by reason for exclusion. We have also referenced this supplementary table in the Results (line 164). |
| 10. Page 10, Table 2: The characteristics table omits columns for key technical parameters, such as the specific evaluation metrics and model temperatures utilized in each study. Expand the table to include these variables, which are mandatory for interpreting algorithmic performance in health contexts. | Temperature and evaluation details for each study are provided in S2 Table. Table 2 summarizes study characteristics and design, and Table 3 shows the outcome domains evaluated. We have also added a summary of temperature reporting to the Results (lines 179–182; see comment 7). To preserve the readability of Table 2, consistent with earlier reviewer feedback, we have retained detailed technical information in S2 Table rather than duplicating it in additional columns. |
| 11. Page 15, Lines 212-219: The synthesis of structural modifications generalizes findings across exercise coaching and medication-use contexts. These domains possess fundamentally different clinical risk profiles. Separate the safety outcome analysis by specific health domain to prevent conflating low-risk lifestyle modifications with high-risk pharmacological guidance. | We have revised the synthesis of structural modifications to distinguish safety findings in medication-use support from those in exercise coaching (lines 220–224). In medication-use support, errors persisted despite improvements in instruction quality; in exercise coaching, structured prompts improved plan quality but safety gaps remained for patients with comorbidities. We have also added quantitative medication-safety findings to the “Outcomes Evaluated” section (lines 294–297), including both decreases and increases in error rates across different prompting comparisons. |
| 12. Page 21, Lines 272-277: The evaluation metrics for output quality currently rely on basic similarity and automated scores without proposing more rigorous computational alternatives. Recommend the adoption of multivariate frameworks and machine learning control designs to rigorously test the temporal and contextual accuracy of automated outputs. Refer to the following citation to propose advanced methodological rigor: [Dhahbi W, Dergaa I: Machine learning with temporal control designs for testing rhythmic specificity in chronobiology: A multivariate framework proposal for distinguishing genuine biological rhythms from temporal artifacts. Chronobiology International 2026:1-8.]. | We have expanded the Discussion to explain the limitations of automated similarity metrics, including that similarity to reference text does not independently establish clinical appropriateness or safety (lines 328–331; see comment 4). The objective of this scoping review was to describe evaluation approaches used in the existing literature, rather than propose or validate new computational frameworks. The suggested article concerns temporal-control designs in chronobiology and does not directly address LLM health coaching or the evaluation of prompt design, so we have not added it. |
| 13. Page 22, Lines 283-288: The user experience domain fails to address the cognitive load and potential for digital distraction present during continuous automated health coaching interactions. Address how these digital health tools might impact cognitive reflection time during mental health interventions. Incorporate the following reference to deepen the user experience discussion: [Dhahbi W, Pyne DB, Dergaa I, Zeitouny D, Müller P, El Omri A, Chamari K, Chaabene H: Science Is About Thinking: How Can We Protect Thinking Time in a Distracted Digital World? Brain Sciences 2026, 16(7):677.]. | None of the included studies measured cognitive load or digital distraction. As this scoping review aims to synthesize outcomes evaluated in the included literature, we have not expanded the discussion to include outcomes beyond the available evidence. The suggested reference by Dhahbi et al. (2026) does not examine AI health coaching chatbots and falls outside the scope of this review, so we have not added it. |
| 14. Page 24, Lines 347-348: The text references “skydiving and other high-risk activities” as an example of unsafe extreme recommendations. This assertion lacks a technical analysis of the initial prompt boundary conditions that permitted this failure. Detail the precise computational or logic constraints missing from the prompt in Grabb et al. to contextualize the algorithmic hallucination. | The example in Grabb et al. should not be interpreted as a factual hallucination: the original prompt explicitly requested extreme and creative suggestions (Table 4). We have revised the text to clarify these instructions and the context of the response (lines 370–373): “For example, when a prompt explicitly instructed the model to act as a non-traditional wellness coach giving extreme and creative suggestions, it recommended skydiving and other high-risk activities.” |
| 15. Page 25, Lines 353-356: The safety outcomes section superficially mentions safety approaches without a deep analysis of the ethical dilemmas and fundamental risks of relying on unstandardized large language model outputs. Expand the safety domain analysis to address the broader dilemma of assisted technology and the specific risks of chatbot hallucinations. Integrate the following reference to support this critique: [Ben Saad H, Dergaa I, Ghouili H, Ceylan Hİ, Chamari K, Dhahbi W: The assisted Technology dilemma: a reflection on AI chatbots use and risks while reshaping the peer review process in scientific research. AI & SOCIETY 2025, 40(7):5649-5656.]. | The manuscript addresses ethical and safety concerns relevant to patient-facing LLM health coaching, including accountability, privacy, bias, and unsafe guidance in the Introduction (lines 79–80), data governance considerations in the Discussion (lines 348–350), and limitations of the predominantly simulation-based safety evidence (lines 381–385). These limitations constrain conclusions about potential real-world harms. As noted in our previous response, Ben Saad et al. (2025) focuses on AI use in scientific peer review rather than patient-facing health coaching. We have therefore retained Fareed et al. (2025), which more directly addresses ethical considerations of LLMs in healthcare. |
