# Supplementary material for: Prompt design in large language model health coaching chatbots: A scoping review
Source: PLOS Digit Health. 2026 Nov 12;5(11):e0001827. doi: 10.1371/journal.pdig.0001827 (PMC13759065; doi:10.1371/journal.pdig.0001827)
Supplement: Peer review history

# Peer review: Decision Letter 0

**DOI:** 10.1371/journal.pdig.0001827.r001

**Authors:** Henry Horng-Shing Lu, Tim M.H. Li

9 Jun 2026

Response to Reviewers Revised Manuscript with Track Changes Manuscript **Journal Requirements:**

1. We note that your Data Availability Statement is currently as follows: All data underlying this scoping review are available within the manuscript.

Please confirm at this time whether or not your submission contains all raw data required to replicate the results of your study. Authors must share the “minimal data set” for their submission. PLOS defines the minimal data set to consist of the data required to replicate all study findings reported in the article, as well as related metadata and methods (https://journals.plos.org/plosone/s/data-availability#loc-minimal-data-set-definition).

For example, authors should submit the following data:

- The values behind the means, standard deviations and other measures reported;

- The values used to build graphs;

- The points extracted from images for analysis.

Authors do not need to submit their entire data set if only a portion of the data was used in the reported study.

If your submission does not contain these data, please either upload them as Supporting Information files or deposit them to a stable, public repository and provide us with the relevant URLs, DOIs, or accession numbers. For a list of recommended repositories, please see https://journals.plos.org/plosone/s/recommended-repositories.

If there are ethical or legal restrictions on sharing a de-identified data set, please explain them in detail (e.g., data contain potentially sensitive information, data are owned by a third-party organization, etc.) and who has imposed them (e.g., an ethics committee). Please also provide contact information for a data access committee, ethics committee, or other institutional body to which data requests may be sent. If data are owned by a third party, please indicate how others may request data access.

2. Please upload separate figure files in .tif or .eps format. Also, remove the figures from your manuscript file but keep the legends.

For more information about figure files please see our guidelines:

https://journals.plos.org/digitalhealth/s/figures

https://journals.plos.org/digitalhealth/s/figures#loc-file-requirements

**Additional Editor Comments (if provided):** **Reviewers' Comments:**

**Comments to the Author**

1. Does this manuscript meet PLOS Digital Health’s publication criteria?

Reviewer #1: Yes

Reviewer #2: Yes

Reviewer #3: Partly

**********

2. Has the statistical analysis been performed appropriately and rigorously?

Reviewer #1: Yes

Reviewer #2: Yes

Reviewer #3: Yes

**********

3. Have the authors made all data underlying the findings in their manuscript fully available (please refer to the Data Availability Statement at the start of the manuscript PDF file)?

The PLOS Data policy

Reviewer #1: Yes

Reviewer #2: Yes

Reviewer #3: Yes

**********

4. Is the manuscript presented in an intelligible fashion and written in standard English?

Reviewer #1: Yes

Reviewer #2: Yes

Reviewer #3: Yes

**********

**Reviewer #1:**  Strengths

1. This review addresses a timely and important topic. Users may follow AI-generated health advice closely, especially in high-stakes situations, which makes prompt design a critical and modifiable factor for safety.

2. The review is methodologically sound, following PRISMA-ScR guidance, with a prospectively registered protocol and independent screening by multiple reviewers.

3. It fills a clear gap, as previous chatbot reviews have not specifically examined prompt design and its impact on response quality and safety.

Weaknesses

1. The three prompt strategies (structural modifications, prompting techniques, and role framing) are repetitive and appear in several place. You could consider trim the redundant descriptions to make the paper flow better.

2. In Figure 1, 1,999 records were excluded at the title/abstract screening stage without reasons provided. The authors should clarify the main reasons for exclusion to improve transparency and allow readers to assess whether relevant studies were inappropriately omitted.

3. The descriptions of the three prompt strategies are presented entirely in prose. The authors should consider adding a table or figure with concrete prompt examples illustrating each strategy, as this would substantially improve clarity for readers unfamiliar with prompt engineering.

4. The size of the datasets used in these studies are not consistently reported in this scoping review. Since dataset size directly affects the reliability and comparability of the reported findings, the authors should extract and report the number of scenarios, dialogues, or records evaluated in each study where applicable.

5. Table 2 currently has limited readability due to frequent line breaks within the 'Prompt design' column. The authors may consider adjusting the column width or font size to improve the visual clarity of this table.

6. The Discussion touches on some limitations of the included studies but these are scattered and not tied together. It would be stronger if the authors pulled these methodological issues together and connected them directly to the future research directions they propose. Right now those directions feel a bit disconnected from the specific weaknesses found across the studies.

**Reviewer #2:**  This manuscript presents a scoping review examining how prompt design has been applied and evaluated in large language model (LLM)-based health coaching chatbots. The authors identified 12 studies and synthesized prompt design approaches into three broad categories: structural modifications, prompting techniques, and role-framing strategies. Outcomes were grouped into output quality, safety, user experience, and clinical outcomes. The review concludes that prompt design is an important determinant of chatbot performance but that current evidence is dominated by simulation-based studies with limited assessment of safety and real-world outcomes.

The topic is timely, relevant, and well aligned with the scope of PLOS Digital Health. Prompt engineering has become a critical issue in the deployment of AI systems in healthcare, and the specific focus on health coaching chatbots addresses an important knowledge gap. The manuscript is generally well written, follows PRISMA-ScR guidance, and provides a useful synthesis of an emerging literature. However, several methodological and reporting issues should be addressed before publication.

Major Strengths

1. Novel and Relevant Research Question

The review addresses an important gap in the literature. While numerous reviews have examined healthcare chatbots and LLM applications, few have focused specifically on prompt design as a determinant of performance. The manuscript clearly articulates this gap and provides a rationale for the review.

2. Appropriate Use of Scoping Review Methodology

The use of a scoping review is justified given the emerging nature of the field. The review follows PRISMA-ScR guidance and includes protocol registration on OSF.

3. Comprehensive Search Strategy

The authors searched five major databases, including both health and engineering sources (MEDLINE, Embase, PsycINFO, IEEE Xplore, and Scopus), which improves coverage of interdisciplinary literature.

4. Useful Conceptual Categorization

The categorization of prompt design strategies into: Structural modifications, Prompting techniques, Role framing provides a practical framework that may be useful for future researchers and developers.

5. Recognition of Safety and Clinical Outcome Gaps

The manuscript appropriately highlights that most studies focus on technical performance while neglecting safety, patient-centered outcomes, and real-world effectiveness. This observation is important and well supported by the findings.

Major Concerns

Limited Discussion of Risk of Bias and Evidence Quality: Although formal risk-of-bias assessment is not mandatory for scoping reviews, the manuscript does not sufficiently discuss the methodological quality of included studies.

The discussion would benefit from a more explicit critique of the strength of evidence underlying the reported findings.

For example:

Which findings are supported by randomized studies?

Which are based solely on simulation?

Which findings should be interpreted cautiously?

Without such distinctions, readers may overestimate the certainty of conclusions. The search Strategy May Miss Relevant Prompt Engineering Literature

The eligibility criteria focus exclusively on "health coaching chatbots."

However, substantial prompt engineering research exists in:

Clinical decision support

Medical question answering

Mental health chatbots

Patient education systems

Digital therapeutics

Many of these studies may provide directly relevant evidence regarding prompt design. The authors should justify why the review was restricted to health coaching and discuss how this restriction may have influenced findings.

Insufficient Analysis of Model-Specific Effects: The review notes that GPT models dominated included studies while a few used LLaMA and DeepSeek models. However, the synthesis largely treats prompt engineering effects as model-independent. A discussion of model-specific variability would strengthen the paper.

Lack of Critical Examination of Safety Evaluations: The manuscript repeatedly notes that safety was assessed in only four studies. However, safety evaluation itself is not critically examined.

Important questions remain unanswered:

Were safety frameworks standardized?

Were harms assessed prospectively or retrospectively?

Were evaluations performed by clinicians?

Were high-risk scenarios tested?

A more detailed discussion is warranted because safety remains one of the major barriers to clinical deployment of LLMs. The review concludes that prompt design matters, but it stops short of providing actionable recommendations. Readers would benefit from a synthesis of emerging best practices such as:

Explicit role assignment

Safety constraints

Structured prompt templates

Retrieval augmentation

Prompt transparency reporting

A practical summary table would increase the translational value of the review. The manuscript reports country distributions but does not discuss implications.

The absence of studies from: Africa, South Asia, Latin America (outside Brazil) should be highlighted because health coaching applications are often intended for diverse populations.

**Reviewer #3:**  General Comments

Major Weaknesses: The primary deficiency of this scoping review is the absence of a standardized assessment of artificial intelligence (AI) reporting quality among the included studies. For a review explicitly focused on "prompt design," it is critical to evaluate whether the primary literature adequately reported large language model (LLM) hyperparameters, exact model versions, and full prompt architectures. The extraction methodology lacks a comprehensive, multi-item reporting checklist to verify the scientific rigor and reproducibility of the underlying data. Furthermore, the categorization of prompt strategies was developed solely through internal discussion without relying on established computational linguistic or machine learning taxonomies.

Minor Weaknesses: There are several typographical errors in the author affiliations section. The PRISMA flow diagram and corresponding text lack detailed stratification of exclusion criteria. Additionally, there is a minor chronological discrepancy regarding the protocol registration and search execution dates that requires clarification.

Specific Comments

Page 1, Lines 5-6: The author affiliations contain typographical errors, specifically the use of periods and missing commas (e.g., "1.8" and "18" instead of superscript "1, 8").

Page 3, Line 79: The discussion of risks and safety concerns of chatbots is overly narrow. The context must be broadened to include the systemic risks and ethical dilemmas of AI chatbot integration in scientific and healthcare applications. Integrate the following reference to support this expansion: [Ben Saad H, Dergaa I, Ghouili H, Ceylan Hİ, Chamari K, Dhahbi W: The assisted Technology dilemma: a reflection on AI chatbots use and risks while reshaping the peer review process in scientific research. AI & SOCIETY 2025, 40(7):5649-5656.]

Page 4, Lines 90-93: The assertion regarding the lack of transparent reporting of prompt design misses a broader, critical point: the general lack of standardized evaluation for AI inputs and outputs across health and performance technologies. Note the calls for standardized assessments of AI tools to support the argument for structured prompt evaluation, referencing: [Dhahbi W, Chamari K: The Algorithmic Athlete: A Call to Standardize Assessment of Sensor Technologies and Artificial Intelligence. International Journal of Sports Physiology and Performance 2026, 1(aop):1-2.]

Page 5, Lines 108-109 & Line 121: There is a chronological inconsistency regarding the prospective registration date of "October 26, 2025" and the search execution date of "October 27, 2025". Clarify how the protocol was adequately prospective if the database search was conducted the subsequent day.

Page 7, Lines 137-139: The data charting process fails to extract critical technical parameters essential for AI reproducibility. The methodology must incorporate a comprehensive reporting checklist to capture whether the included studies detailed exact LLM versions, temperature settings, context window limits, and prompt token counts.

Page 8, Lines 144-149: The conceptual categories for prompt design strategies (structural modifications, prompting techniques, role framing) are not grounded in established machine learning literature. Provide a theoretical justification or cite existing computer science frameworks to validate this taxonomy.

Page 10, Lines 159-160: The PRISMA flow diagram groups 75 excluded studies under a single "Wrong intervention" label. Stratify this number to specify how many were excluded for lacking LLMs, lacking a chatbot interface, or lacking the health coaching element to improve methodological transparency.

Page 11-14, Table 2: The "Prompt design" column provides generic summaries rather than actionable data. Extract and present specific verbatim prompt examples or structural templates from the primary studies to improve the utility of this synthesis.

Page 17, Lines 212-217: The definitions of specific prompting techniques (e.g., zero-shot, few-shot, chain-of-thought, retrieval-augmented generation) lack supporting citations from the foundational AI literature. Integrate primary computational references for these concepts.

Page 21, Lines 294-298: The discussion of structural modifications omits an analysis of data privacy and data governance. Address the implications of embedding sensitive physiological/behavioral parameters or strict clinical guidelines within prompts processed by proprietary, cloud-based LLMs.

Page 21, Lines 297-302: The comparison between structural modifications and prompting techniques for health coaching lacks depth regarding how AI-generated guidance contrasts with actual human-led dialogue. Contrast these findings with existing literature evaluating AI-based guidance against human coaching to highlight the nuanced differences in reflective problem-solving. Cite the following reference: [Sahli H, Bouzouraa MM, Rebhi M, Ceylan Hİ, Yılmaz HH, Ovidiu D, Muntean RI, Dhahbi W, Naima A, Zghibi M: Comparing human-led reflective dialogue and AI-based tactical guidance as innovative training approaches in soccer: effects on tactical performance and problem-solving. BMC Sports Science, Medicine and Rehabilitation 2026, 18(1):246.]

Page 23, Lines 338-339: The limitations section does not address the high risk of publication bias and the rapid obsolescence of findings. Address this limitation directly, as the majority of the included literature evaluates models that are rapidly superseded by newer architectures.

Page 23, Lines 346-349: The conclusion's call for standardized evaluation frameworks and real-world studies is vague. Provide actionable direction by recommending the adoption of modular frameworks for the development and validation of autonomous AI agents to ensure safety and clinical alignment. Support this recommendation by citing: [Dergaa I, Barbaria S, Dhahbi W, Zmijewski P, Chamari K, Saad HB: Toward autonomous artificial intelligence agents in sports science: a modular framework for development, validation, and implementation. Biology of Sport 2026, 43(1):1353-1426.]

**********

what does this mean?). If published, this will include your full peer review and any attached files.

**Do you want your identity to be public for this peer review?**  If you choose “no”, your identity will remain anonymous but your review may still be made public.

For information about this choice, including consent withdrawal, please see our Privacy Policy

Reviewer #1: **Yes:**  Renjie Liang

Reviewer #2: No

Reviewer #3: **Yes:**  Wissem Dhahbi

**********

**Figure resubmission:**

**Reproducibility:**

To enhance the reproducibility of your results, we recommend that authors of applicable studies deposit laboratory protocols in protocols.io, where a protocol can be assigned its own identifier (DOI) such that it can be cited independently in the future. Additionally, PLOS ONE offers an option to publish peer-reviewed clinical study protocols. Read more information on sharing protocols at https://plos.org/protocols?utm_medium=editorial-email&utm_source=authorletters&utm_campaign=protocols

# Author comment: Author response to Decision Letter 1

**DOI:** 10.1371/journal.pdig.0001827.r002

24 Jul 2026

Attachment Submitted filename: Response to Reviewers.docx

# Peer review: Decision Letter 1

**DOI:** 10.1371/journal.pdig.0001827.r003

**Authors:** Henry Horng-Shing Lu, Tim M.H. Li

25 Aug 2026

Response to ReviewersRevised Manuscript with Track ChangesManuscript**Journal Requirements:** **Additional Editor Comments (if provided):** **Reviewers' Comments:**

**Comments to the Author**

Reviewer #1: All comments have been addressed

Reviewer #2: All comments have been addressed

Reviewer #3: All comments have been addressed

**********

publication criteria?

Reviewer #1: Yes

Reviewer #2: Yes

Reviewer #3: Partly

**********

3. Has the statistical analysis been performed appropriately and rigorously?

Reviewer #1: Yes

Reviewer #2: Yes

Reviewer #3: Yes

**********

4. Have the authors made all data underlying the findings in their manuscript fully available (please refer to the Data Availability Statement at the start of the manuscript PDF file)?

The PLOS Data policy

Reviewer #1: Yes

Reviewer #2: Yes

Reviewer #3: Yes

**********

5. Is the manuscript presented in an intelligible fashion and written in standard English?

Reviewer #1: Yes

Reviewer #2: Yes

Reviewer #3: Yes

**********

**Reviewer #1:**  The authors have addressed all of my previous comments. The revised manuscript is clearer and better organized. The added prompt examples and sample-size information are helpful, and the Discussion now better connects the limitations of the included studies with directions for future research. I have no further comments.

**Reviewer #2:**  (No Response)

**Reviewer #3:**  General Comments

The revised scoping review provides a comprehensive synthesis of prompt design strategies in health coaching chatbots. The major weakness of the current version is the introduction of numerous typographical and grammatical errors during the revision process, particularly where tracked changes appear to have been improperly merged within the manuscript text. The minor weakness involves a few lingering inconsistencies in formatting and spacing throughout the results and discussion sections.

Scientific Content, Statistics, and Methodology

The scientific content remains relevant and logically structured, successfully mapping the current evidence base regarding prompt design. The methodology strictly adheres to PRISMA-ScR guidelines, and the narrative synthesis is appropriate for the extracted data. Since this is a scoping review, formal statistical hypothesis testing is not applicable, but the descriptive reporting of study characteristics is accurate. The writing quality requires attention to correct the mechanical errors introduced in this revision round.

Specific Comments

Regarding the Methods section, on Page 8, Line 150, there is a typographical error with the text "tructural Structural modifications". On Page 9, Line 152, correct the typographical error "Pprompting techniques". On Page 9, Line 153, correct the typographical error "Rand-role framing strategies".

Within the Results section, on Page 11, Line 177, the model name contains a duplication error appearing as "LlamaLLaMA-3/3.1". On Page 15, Line 201, correct the spelling error "empireallyempirically". On Page 23, Line 288, fix the spacing and punctuation error reading "unsafe Safetyoutputs25, Safety was evaluated".

For the Discussion section, on Page 25, Line 334, correct the formatting issue in the phrase "warrant Promptingattention". On Page 25, Line 336, remove the unnecessary hyphen in "impact-tone". On Page 27, Line 387, correct the typographical error "and-highlighted". On Page 28, Lines 399 to 400, the sentence beginning with "Finallyresearch" is garbled and contains multiple errors, including "hhealth" and "contextss", requiring a complete rewrite for clarity.

**********

what does this mean?). If published, this will include your full peer review and any attached files.

**Do you want your identity to be public for this peer review?**  If you choose “no”, your identity will remain anonymous but your review may still be made public.

For information about this choice, including consent withdrawal, please see our Privacy Policy

Reviewer #1: **Yes:**  Renjie Liang

Reviewer #2: No

Reviewer #3: **Yes:**  Wissem Dhahbi

**********

**Figure resubmission:**

**Reproducibility:**

To enhance the reproducibility of your results, we recommend that authors of applicable studies deposit laboratory protocols in protocols.io, where a protocol can be assigned its own identifier (DOI) such that it can be cited independently in the future. Additionally, PLOS ONE offers an option to publish peer-reviewed clinical study protocols. Read more information on sharing protocols at https://plos.org/protocols?utm_medium=editorial-email&utm_source=authorletters&utm_campaign=protocols

# Author comment: Author response to Decision Letter 2

**DOI:** 10.1371/journal.pdig.0001827.r004

15 Sep 2026

Attachment Submitted filename: Response to Reviewer Comments.docx

# Peer review: Decision Letter 2

**DOI:** 10.1371/journal.pdig.0001827.r005

**Authors:** Henry Horng-Shing Lu, Tim M.H. Li

24 Sep 2026

Response to ReviewersRevised Manuscript with Track ChangesManuscript**Journal Requirements:** **Additional Editor Comments (if provided):** **Reviewers' Comments:**

**Comments to the Author**

Reviewer #3: All comments have been addressed

**********

publication criteria?

Reviewer #3: Partly

**********

3. Has the statistical analysis been performed appropriately and rigorously?

Reviewer #3: Yes

**********

4. Have the authors made all data underlying the findings in their manuscript fully available (please refer to the Data Availability Statement at the start of the manuscript PDF file)?

The PLOS Data policy

Reviewer #3: Yes

**********

5. Is the manuscript presented in an intelligible fashion and written in standard English?

Reviewer #3: Yes

**********

Reviewer #3: General Comments

The manuscript presents a scoping review examining prompt design strategies in large language model health coaching chatbots. A major weakness of the manuscript is the absence of a structured quality appraisal of the included studies. While optional in scoping reviews, critical appraisal is strictly necessary when assessing experimental artificial intelligence applications in clinical or sports medicine contexts to distinguish between rigorous methodological frameworks and anecdotal observations. The synthesis relies heavily on the descriptive categorization of structural modifications, prompting techniques, and role framing, yet it fails to rigorously map how these prompt architectures interact with specific physiological or behavioral outcomes. A minor weakness is the inconsistent integration of extracted variables. The methodology states that technical parameters such as model temperature were extracted, but these variables are largely absent from the results synthesis and study characteristics table.

Scientific Content, Methodology, and Statistics

The methodological framework adheres to the PRISMA-ScR guidelines and includes a prospectively registered protocol. The search strategy encompasses appropriate databases for the intersection of computer science and health, including IEEE Xplore and PubMed. The authors correctly omitted statistical meta-analysis due to the heterogeneity of the 12 included simulation-based studies. The narrative synthesis lacks a professional biomedical engineering or biostatistical perspective on how natural language processing evaluation metrics are quantified. The categorization of outcomes into output quality, safety, user experience, and clinical outcomes is logical, but the manuscript does not critically evaluate the statistical validity of the automated similarity metrics or participant-reported measures utilized in the primary studies. Relying on superficial descriptions of evaluation metrics limits the academic rigor required for artificial intelligence integration in health behavior change protocols.

Specific Comments

Page 5, Line 106: The introduction abruptly concludes the rationale for the scoping review without contextualizing how artificial intelligence prompt design methodologies compare to broader automated applications in health and personalized prevention. Broaden the discussion on methodological frameworks used in scoping reviews within health contexts to provide a comparative baseline for evaluating structural modifications. Include the following reference to strengthen this context: [Dhahbi W, Jebabli N, Souaifi M, Ceylan Hİ, Ben Saad H, Chamari K, Pyne DB, Chaabene H: Application of Artificial Intelligence for Predicting Sports Injuries and Customizing Personalized Prevention Strategies: A Scoping Review. Bioengineering 2026, 13(6):692.].

Page 7, Lines 123-124: The search period is defined as extending to October 27, 2025. The reference list includes publications dated 2026, such as Njei et al., 2026, and de Haan et al., 2026. Correct the chronological boundaries of the search strategy and align the inclusion dates with the bibliography.

Page 7, Lines 137-142: The data charting process explicitly extracts "temperature" and "sample size". These critical technical metrics are excluded from the primary results text and Table 2. Integrate all charted artificial intelligence parameters into the final synthesis to ensure reproducibility.

Page 8, Lines 148-154: The taxonomy of prompt design strategies defines structural modifications, prompting techniques, and role framing. The methodology lacks a transparent mapping of how complex prompts containing multiple overlapping elements were isolated and categorized. Provide a clear methodological protocol for handling hybrid prompt designs.

Page 9, Line 165: The PRISMA flow diagram indicates 81 full-text articles were excluded. The manuscript fails to provide a categorized breakdown of exclusion reasons beyond the broad numerical summary in the diagram. Add a supplementary table detailing the specific rationales for full-text exclusions.

Page 10, Table 2: The characteristics table omits columns for key technical parameters, such as the specific evaluation metrics and model temperatures utilized in each study. Expand the table to include these variables, which are mandatory for interpreting algorithmic performance in health contexts.

Page 15, Lines 212-219: The synthesis of structural modifications generalizes findings across exercise coaching and medication-use contexts. These domains possess fundamentally different clinical risk profiles. Separate the safety outcome analysis by specific health domain to prevent conflating low-risk lifestyle modifications with high-risk pharmacological guidance.

Page 21, Lines 272-277: The evaluation metrics for output quality currently rely on basic similarity and automated scores without proposing more rigorous computational alternatives. Recommend the adoption of multivariate frameworks and machine learning control designs to rigorously test the temporal and contextual accuracy of automated outputs. Refer to the following citation to propose advanced methodological rigor: [Dhahbi W, Dergaa I: Machine learning with temporal control designs for testing rhythmic specificity in chronobiology: A multivariate framework proposal for distinguishing genuine biological rhythms from temporal artifacts. Chronobiology International 2026:1-8.].

Page 22, Lines 283-288: The user experience domain fails to address the cognitive load and potential for digital distraction present during continuous automated health coaching interactions. Address how these digital health tools might impact cognitive reflection time during mental health interventions. Incorporate the following reference to deepen the user experience discussion: [Dhahbi W, Pyne DB, Dergaa I, Zeitouny D, Müller P, El Omri A, Chamari K, Chaabene H: Science Is About Thinking: How Can We Protect Thinking Time in a Distracted Digital World? Brain Sciences 2026, 16(7):677.].

Page 24, Lines 347-348: The text references "skydiving and other high-risk activities" as an example of unsafe extreme recommendations. This assertion lacks a technical analysis of the initial prompt boundary conditions that permitted this failure. Detail the precise computational or logic constraints missing from the prompt in Grabb et al. to contextualize the algorithmic hallucination.

Page 25, Lines 353-356: The safety outcomes section superficially mentions safety approaches without a deep analysis of the ethical dilemmas and fundamental risks of relying on unstandardized large language model outputs. Expand the safety domain analysis to address the broader dilemma of assisted technology and the specific risks of chatbot hallucinations. Integrate the following reference to support this critique: [Ben Saad H, Dergaa I, Ghouili H, Ceylan Hİ, Chamari K, Dhahbi W: The assisted Technology dilemma: a reflection on AI chatbots use and risks while reshaping the peer review process in scientific research. AI & SOCIETY 2025, 40(7):5649-5656.].

**********

what does this mean?). If published, this will include your full peer review and any attached files.

**Do you want your identity to be public for this peer review?**  If you choose “no”, your identity will remain anonymous but your review may still be made public.

For information about this choice, including consent withdrawal, please see our Privacy Policy

Reviewer #3: **Yes:**  Wissem Dhahbi

**********

**Figure resubmission:**

**Reproducibility:**

To enhance the reproducibility of your results, we recommend that authors of applicable studies deposit laboratory protocols in protocols.io, where a protocol can be assigned its own identifier (DOI) such that it can be cited independently in the future. Additionally, PLOS ONE offers an option to publish peer-reviewed clinical study protocols. Read more information on sharing protocols at https://plos.org/protocols?utm_medium=editorial-email&utm_source=authorletters&utm_campaign=protocols

# Author comment: Author response to Decision Letter 3

**DOI:** 10.1371/journal.pdig.0001827.r006

24 Oct 2026

Attachment Submitted filename: Response_to_Reviewers.docx

# Peer review: Decision Letter 3

**DOI:** 10.1371/journal.pdig.0001827.r007

**Authors:** Henry Horng-Shing Lu, Tim M.H. Li

28 Oct 2026

Prompt design in large language model health coaching chatbots: a scoping review

PDIG-D-26-00737R3

Dear Ms. Incze,

We are pleased to inform you that your manuscript 'Prompt design in large language model health coaching chatbots: a scoping review' has been provisionally accepted for publication in PLOS Digital Health.

Before your manuscript can be formally accepted you will need to complete some formatting changes, which you will receive in a follow-up email from a member of our team.

Please note that your manuscript will not be scheduled for publication until you have made the required changes, so a swift response is appreciated.

IMPORTANT: The editorial review process is now complete. PLOS will only permit corrections to spelling, formatting or significant scientific errors from this point onwards. Requests for major changes, or any which affect the scientific understanding of your work, will cause delays to the publication date of your manuscript.

If your institution or institutions have a press office, please notify them about your upcoming paper to help maximize its impact. If they'll be preparing press materials, please inform our press team as soon as possible -- no later than 48 hours after receiving the formal acceptance. Your manuscript will remain under strict press embargo until 2 pm Eastern Time on the date of publication. For more information, please contact digitalhealth@plos.org.

Thank you again for supporting Open Access publishing; we are looking forward to publishing your work in PLOS Digital Health.

Best regards,

Tim M.H. Li, PhD

Section Editor

PLOS Digital Health

***********************************************************

**Additional Editor Comments (if provided):**

**Reviewer Comments (if any, and for reference):**

Reviewer's Responses to Questions

**Comments to the Author**

Reviewer #3: All comments have been addressed

**********

publication criteria?

Reviewer #3: Yes

**********

3. Has the statistical analysis been performed appropriately and rigorously?

Reviewer #3: Yes

**********

4. Have the authors made all data underlying the findings in their manuscript fully available (please refer to the Data Availability Statement at the start of the manuscript PDF file)?

The PLOS Data policy

Reviewer #3: Yes

**********

5. Is the manuscript presented in an intelligible fashion and written in standard English?

Reviewer #3: Yes

**********

**Reviewer #3:**  The manuscript presents a rigorous and well-structured scoping review examining prompt design strategies within large language model health coaching chatbots. The authors have undertaken substantial revisions that directly address previous methodological critiques, thereby enhancing the overall clarity, transparency, and scholarly merit of the work. The inclusion of supplementary documentation, such as detailed exclusion rationales and expanded descriptions of technical parameters including temperature settings and sample sizes, significantly improves the reproducibility and transparency of the review. Furthermore, the narrative synthesis successfully delineates between diverse health domains, providing a clearer distinction regarding safety outcomes in medication support compared with physical activity guidance.

**********

what does this mean?). If published, this will include your full peer review and any attached files.

**Do you want your identity to be public for this peer review?**  If you choose “no”, your identity will remain anonymous but your review may still be made public.

For information about this choice, including consent withdrawal, please see our Privacy Policy

Reviewer #3: **Yes:**

**********
